# Creating high-resolution 3D cranial implant geometry using deep learning techniques

**DOI:** 10.3389/fbioe.2023.1297933

**Published:** 2023-12-11

**Authors:** Chieh-Tsai Wu, Yao-Hung Yang, Yau-Zen Chang

**Affiliations:** ^1^ Department of Neurosurgery, Linkou Chang Gung Memorial Hospital, Taoyuan, Taiwan; ^2^ College of Medicine, Chang Gung University, Taoyuan, Taiwan; ^3^ ADLINK Technology, Inc, Taoyuan, Taiwan; ^4^ Department of Mechanical Engineering, Chang Gung University, Taoyuan, Taiwan; ^5^ Department of Mechanical Engineering, Ming Chi University of Technology, New Taipei City, Taiwan

**Keywords:** cranioplasty, cranial implant, deep learning, defective skull models, volumetric resolution, 3D inpainting

## Abstract

Creating a personalized implant for cranioplasty can be costly and aesthetically challenging, particularly for comminuted fractures that affect a wide area. Despite significant advances in deep learning techniques for 2D image completion, generating a 3D shape inpainting remains challenging due to the higher dimensionality and computational demands for 3D skull models. Here, we present a practical deep-learning approach to generate implant geometry from defective 3D skull models created from CT scans. Our proposed 3D reconstruction system comprises two neural networks that produce high-quality implant models suitable for clinical use while reducing training time. The first network repairs low-resolution defective models, while the second network enhances the volumetric resolution of the repaired model. We have tested our method in simulations and real-life surgical practices, producing implants that fit naturally and precisely match defect boundaries, particularly for skull defects above the Frankfort horizontal plane.

## 1 Introduction

Skull defects can arise from various causes, including trauma, congenital malformations, infections, and iatrogenic treatments such as decompressive craniectomy, plastic surgery, and tumor resection. Recent studies (Yeap et al., 2019; Alkhaibary et al., 2020) have demonstrated that reconstructing extensive skull defects can significantly improve patients’ physiological and neurological processes by restoring cerebrospinal fluid dynamics and motor and cognitive functions. However, designing a customized implant for cranioplasty is complex and expensive, especially in cases with comminuted fractures.

Advances in medical imaging and computational modeling have enabled the creation of custom-made implants using computer-aided design software. The design process typically involves intensive human-machine interaction using specialized software and requires medical expertise. For example (Lee et al., 2009; Chen et al., 2017), have used mirrored geometry as a starting point for developing an implant model. However, since most human skulls are asymmetric to the sagittal plane, a unilateral defect may still require significant modification to fit the defect boundary after the mirroring operation, let alone defects spanning both sides.

Significant progress has been made in deep learning-based 2D image restoration. For instance (Yang et al., 2017), proposed a multi-scale convolutional neural network to provide high-frequency details for defect reconstruction. The image inpainting schemes of (Pathak et al., 2016; Iizuka et al., 2017) used an encoder-decoder network structure (Hinton and Salakhutdinov, 2006; Baldi, 2011; Dai et al., 2017) for adversarial loss training based on the Generative Adversarial Networks scheme (Goodfellow et al., 2014; Li et al., 2017). Yan and coauthors (Yan et al., 2018) also introduced a shift connection layer in the U-Net architecture (Ronneberger et al., 2015) for repairing defective images with fine details.

While deep learning techniques have made noteworthy progress in 2D image completion, 3D shape inpainting remains challenging due to the higher dimensionality and computational requirements to process 3D data (Maturana and Scherer, 2015). Among the early studies, Morais and coauthors in (Morais et al., 2019) conducted a pioneering study using an encoder-decoder network to reconstruct defective skull models at a volumetric resolution of up to 120 × 120 × 120 by integrating eight equally sized voxel grids of size 60 × 60 × 60.

In (Mahdi, 2021), a U-net (Ronneberger et al., 2015) scheme was developed to predict complete skulls, where the cropped skull modes were down-sampled and rescaled to a voxel resolution of 192 × 256 × 128. This investigation also demonstrates the importance of the quantity and diversity of datasets to ensure the quality and robustness of network predictions (Li et al., 2021a). proposed a patch-based training strategy for 3D shape completion by assembling an encoder-decoder network and a U-net on 128 × 128 × 128 patches cropped from defective skull models. This approach alleviates the memory and computational power requirements. However, when the size of the defect is close to the patch size, the reconstruction performance significantly worsens. Besides, as observed in (Li et al., 2021b), merging patches can easily lead to uneven surfaces.

In (Ellis and Aizenberg, 2020), four 3D U-Net (Ronneberger et al., 2015) models with the same architecture were trained separately in an ensemble. All four models were used to predict complete skulls with a volume resolution of 176 × 224 × 144, and the results were averaged as the final output. The paper reported the loss of edge voxels at the corners of implants. Matzkin and coauthors in (Matzkin et al., 2020a) also used the U-Net architecture for 3D skull model reconstruction and concluded that estimating the implant directly may produce less noise. In a follow-up work by Matzkin and coauthors in (Matzkin et al., 2020b), a shape constructed by averaging healthy head CT images is concatenated with the input to provide complementary information to facilitate the robustness of the model predictions.

Besides, the Statistical Shape Modeling technique (SSM) (Fuessinger et al., 2019; Xiao et al., 2021) can model 3D shapes explicitly from a collection of datasets. This method is inherently insensitive to defect size and shape and can potentially reconstruct skull defects (Li et al., 2022). demonstrated its application to substantial and complex defects, but this approach performed worse on medium-sized synthetic defects than deep learning-based methods.

More recently, Wu and coauthors (Wu et al., 2022) successfully developed a dilated U-Net for 3D skull model reconstruction with a volumetric resolution of 112 × 112 × 40. However, the repairable defect area was limited to the upper parts of skulls, and the voxel resolution was insufficient for direct use in implant fabrication. Building on this work, we propose a new approach to advance the 3D skull model inpainting technique in this paper. Our approach can reconstruct skull models with a higher volumetric resolution of 512 × 512 × 384, meeting the needs of cranial implant design.

**FIGURE 1:**
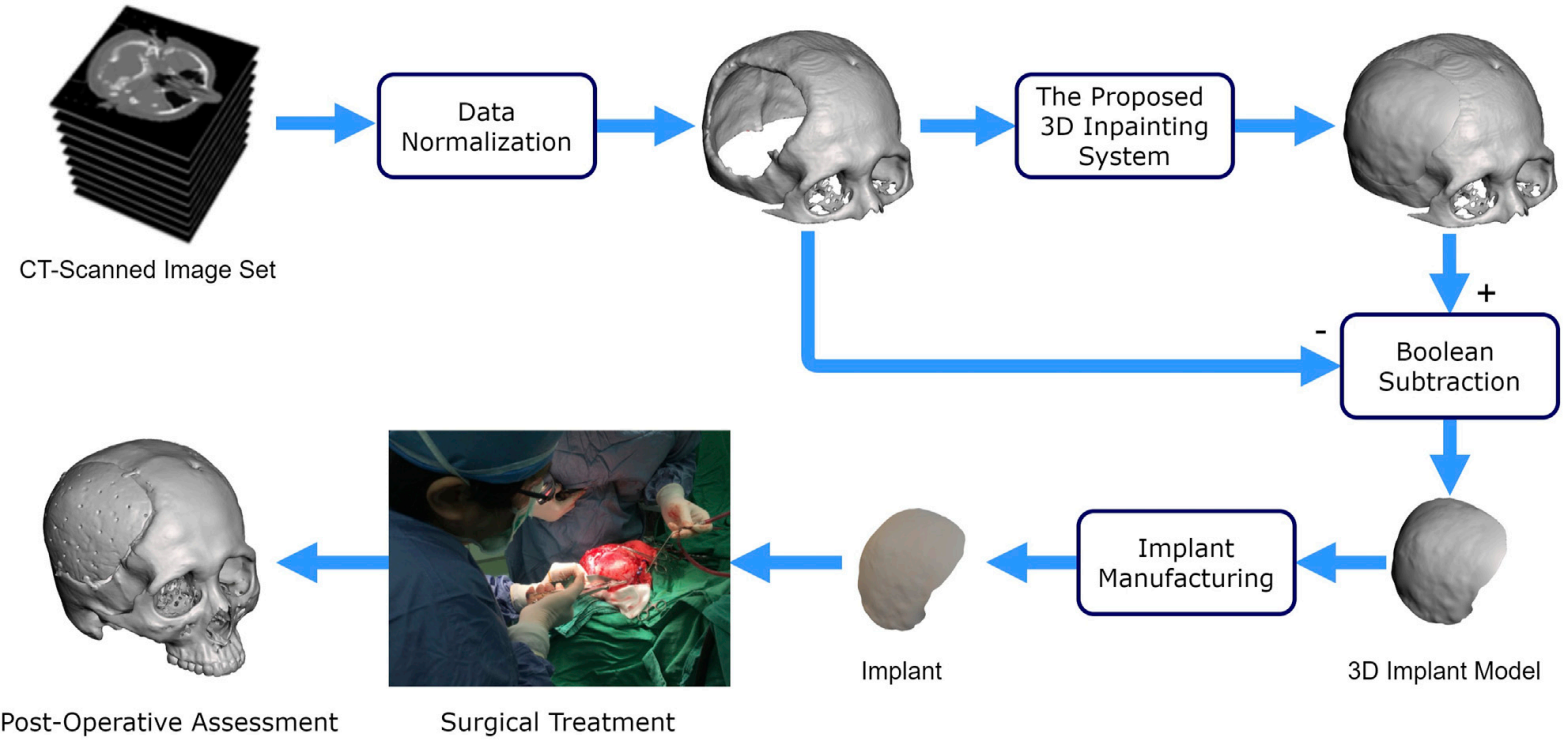
Flowchart of cranial restoration using the proposed deep learning technique.

Figure 1 illustrates the use of our proposed deep learning system for cranioplasty. The system inputs a normalized defective 3D skull model derived from a set of CT-scanned images. Using this defective model, our system automatically reconstructs the skull model. A 3D implant model is then obtained by subtracting the original defective model from the completed model. Once a validated implant model is ready, technicians can use manufacturing processes such as 3D printing and molding (Lee et al., 2009; Wu et al., 2021) to convert raw materials into an implant for surgical treatment.

## 2 Materials and methods

The effectiveness of a deep learning system relies on several factors, including the quality of training data, the network architecture, and training strategies. In this section, we delve into these aspects in detail.

### 2.1 Skull dataset

We collected and curated a dataset of skull models to train and evaluate neural networks. This dataset includes pairs of intact and defective skull models, with the defective models created by applying 3D masks to the intact ones. These skull models were carefully selected from three datasets described below.

#### 2.1.1 Publicly available datasets

The binary datasets, SkullFix (Li and Egger, 2020; Kodym et al., 2021) and SkullBreak (Li and Egger, 2020), were derived from an open-source collection of head-CT images known as the CQ500 dataset (Chilamkurthy et al., 2018). The SkullFix dataset was released for the first MICCAI AutoImplant Grand Challenge (Li and Egger, 2020) in 2020, while the SkullBreak dataset was provided for the second MICCAI AutoImplant Challenge (Li and Egger, 2020) in 2021.

In these datasets, defective models were created by masking certain areas of intact 3D skull models. SkullFix defects are circular or rectangular, while SkullBreak defects are more irregular to mimic traumatic skull fractures. In this study, we selected only 92 intact models from these two datasets.

#### 2.1.2 A retrospective dataset

The Department of Neurosurgery, Chang Gung Memorial Hospital, Taiwan, gathered a dataset over the last 12 years. To ensure confidentiality, the Institutional Review Board, Chang Gung Medical Foundation, Taiwan, under the number 202002439B0, approved removing sensitive information about individuals. Out of the 343 sets of collected data, only 75 datasets were used in this study due to incompleteness or the presence of bone screws. Since image acquisition conditions vary, the bone density of each patient is also different, which necessitated setting the intensity threshold for extracting bone tissue individually, generally within the Hounsfield scale interval [1200, 1817].

During our research, we simplified the skull models we had gathered to reduce memory usage. This was accomplished by removing the bone tissue below the Frankfort horizontal plane (Pittayapat et al., 2018). Besides, although CT images typically have a planar resolution of 512 × 512 pixels, the slice interval can vary from 0.3 to 1.25 mm. To ensure a consistent volumetric resolution of 512 × 512 × 384 voxels, we used the Lanczos interpolation method (Mottola et al., 2021) to resample the skull datasets in the craniocaudal direction. As a result, the cranial models had a typical voxel size of 0.45 mm × 0.45 mm × 0.8 mm.

**FIGURE 2:**
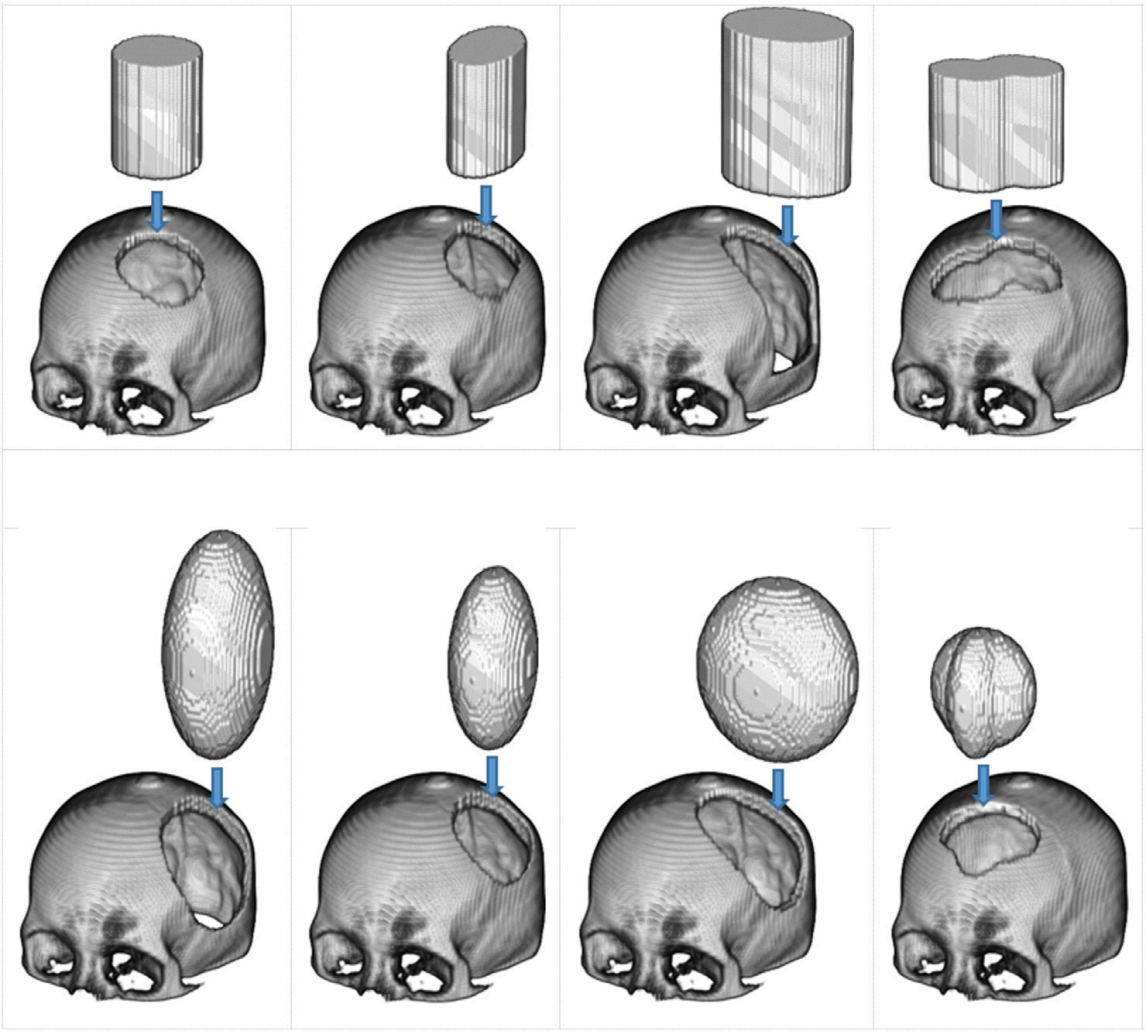
The use of 3D masks to generate simulated defects on cranial models. In the top row are two types of masks: the elliptical cylinder and the mixed elliptical cylinder. The bottom row, on the other hand, has two different mask types: the ellipsoid and the hybrid ellipsoid.

As shown in Figure 2, defects were created on complete skull models using elliptical-cylindrical or ellipsoidal 3D masks to produce a diverse training dataset with different sizes and shapes of defects. The masks were applied randomly to various positions on the skull model, ranging from 60 to 120 mm in diameter. Twenty-five defect variations were injected into each complete skull model.

We employed a data augmentation technique (Shorten and Khoshgoftaar, 2019) to expand the skull dataset by rotating the skull models along the craniocaudal axis. The rotation interval was set at 2°, resulting in seven variants for each skull model. Notably, three variants were generated on one side.

Eventually, we collected 25,930 datasets of paired skull models after removing models with out-of-range defects. Each dataset comprises two intact skull models and two defective ones, which were normalized to two volumetric resolutions: 512 × 512 × 384 and 128 × 128 × 96. The lower-resolution model was down-sampled from the corresponding higher-resolution model. Our final skull data was divided into three groups: training data comprising 21,600 datasets, validation data of 2,400 datasets, and test data of 1,930 datasets.

### 2.2 3D completion and resolution enhancement network architectures

**FIGURE 3:**
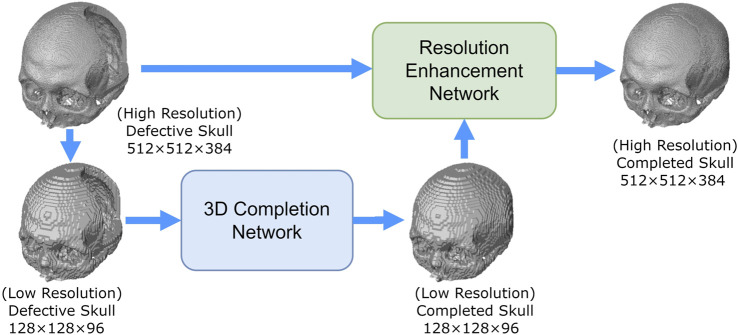
Overview of the proposed 3D inpainting system. The system comprises two networks: a 3D completion network and a resolution enhancement network. The 3D completion network completes the defective skull model with a volume resolution of 128 × 128×96. The resolution enhancement network uses both the completed model of 128 × 128 × 96 and the defective model of 512 × 512 × 384 to generate a 512 × 512 × 384 completed skull model.

We developed two deep-learning networks to predict complete skull models from incomplete ones. As shown in Figure 3, the first is a 10-layer 3D completion network, and the second is a 14-layer resolution enhancement network. Both networks were trained using a supervised learning approach on the training dataset of two different volumetric resolutions.

A defective skull model is first normalized to a volume resolution of 512 × 512 × 384 to prepare input for the networks, retaining only the bone tissue above the Frankfort horizontal plane (Pittayapat et al., 2018). This normalized model is then transformed into a low-resolution defective cranial model with a resolution of 128 × 128 × 96, which becomes the input for the 3D completion network. The network predicts a 128 × 128 × 96 completed skull model. By downsampling the 3D skull model, the computational resources required to process the data are reduced. Finally, the 3D resolution enhancement network uses both the 128 × 128 × 96 completed skull model and the original 512 × 512 × 384 defective skull model as inputs to generate a 512 × 512 × 384 completed skull model.

#### 2.2.1 The 3D completion network

**FIGURE 4:**
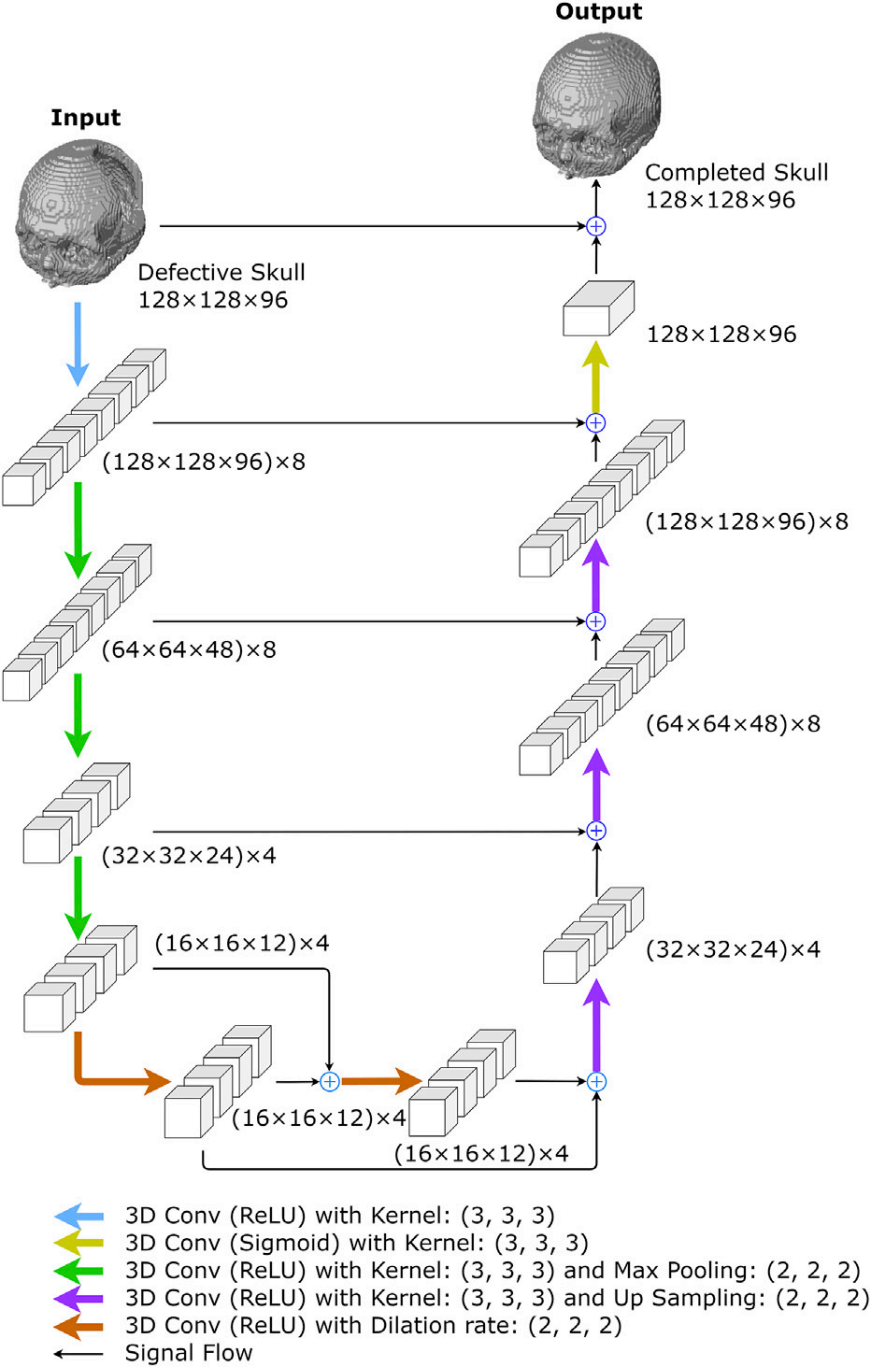
The proposed 3D completion network. The network was created to reconstruct low-resolution skull models. The numbers represent the size of I/O data or feature maps, i.e., the outputs of convolutional layers. For example, (128 × 128 × 96) x 8 describes an 8-channel tensor of size 128 × 128 × 96 in each channel.

This network uses a 3D U-Net (Ronneberger et al., 2015) with 3D dilations at the bottleneck section, as illustrated in Figure 4 and Table 1. The network employs 3 × 3 × 3 kernels in all convolutional layers, including basic convolutions and dilated convolutions, with a dilation rate of 2 for all dilated convolutions. Additionally, all max-pooling operators are of size 2 × 2 × 2.

**TABLE 1:** The architecture of the 10-layer 3D completion network. “AF” is the activation function succeeding a convolutional layer and “Channels” depicts the filter number of a convolutional layer. All convolutional kernels in the network are of size 3 × 3×3 with stride = 1.

Type	Dilation	AF	Channels
3D Convolution	1	ReLU	8
3D Convolution + Max Pooling	1	ReLU	8
3D Convolution + Max Pooling	1	ReLU	4
3D Convolution + Max Pooling	1	ReLU	4
Dilated 3D Convolution	2	ReLU	4
Dilated 3D Convolution	2	ReLU	4
3D Up-Sampling	1	ReLU	4
3D Up-Sampling	1	ReLU	8
3D Up-Sampling	1	ReLU	8
3D Convolution	1	Sigmoid	1

The network begins with a convolution layer and rectified linear unit (ReLU) activations (Agarap, 2018), generating an 8-channel feature map. The down-sampling section on the left side of the network repeatedly performs three convolutions, followed by ReLU activations and max-pooling operations. After each convolution operation, the size of feature maps is halved, while the number of channels remains at 8, 4, and 4, respectively. The bottleneck comprises two dilated convolutional layers with four filters, each connected by skip-connections (Alkhaibary et al., 2020) and followed by ReLU activations (Agarap, 2018).

In the up-sampling section, there are more up-convolutions followed by ReLU activations, and the corresponding feature maps from the down-sampling section are added in. We used nearest-neighbor interpolation upsampling (Kolarik et al., 2019) for the up-convolutions, which assigns the grayscale value from the nearest original voxel to each new voxel.

After that, a convolution layer with sigmoid activation functions is applied to the feature maps from the up-sampling path. The final network output is obtained by adding the result to the original network input. The 3D completion network is made up of 8,269 trainable parameters.

#### 2.2.2 The 3D resolution enhancement network

**FIGURE 5:**
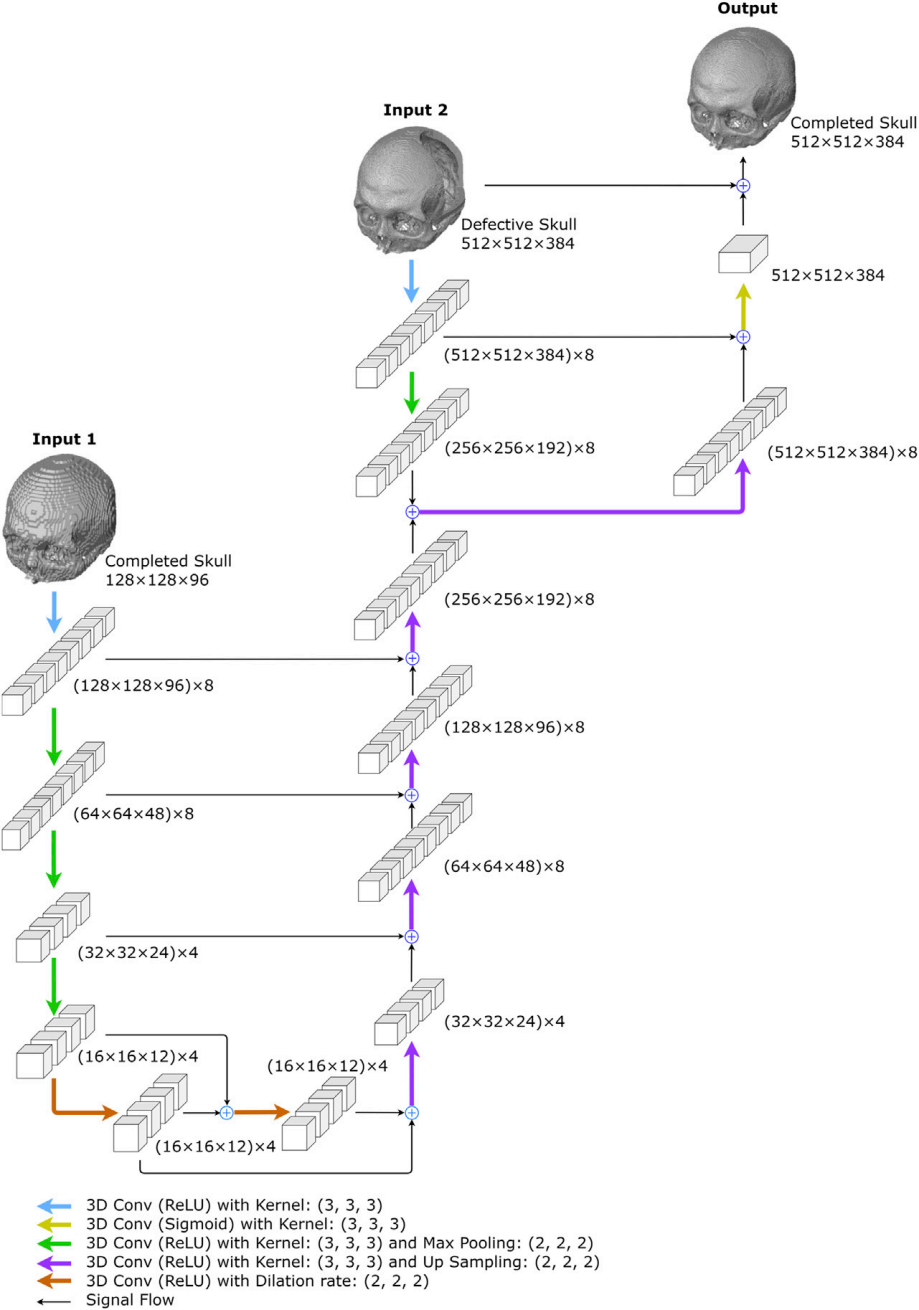
The proposed resolution enhancement network. The network was created to predict high-resolution completed skull models. Two inputs are required: the low-resolution completed skull model created in the first stage and the original high-resolution defective model.

The network predicts a high-resolution completed skull model using a low-resolution completed model and a high-resolution defective model. As shown in Figure 5 and Table 2, the network combines a 3D completion network and a shallower U-Net (Ronneberger et al., 2015). The 3D completion architecture provides a geometric abstraction of the complete low-resolution model to enhance the high-resolution defective model. All convolutional filter kernels and max-pooling operators in the network are 3 × 3 × 3 and 2 × 2 × 2, respectively, similar to the 3D completion network.

**TABLE 2:** The architecture of the 14-layer resolution enhancement network. “AF” is the activation function succeeding a convolutional layer and “Channels” depicts the filter number of a convolutional layer. All convolutional kernels in the network are of size 3 × 3×3 with stride = 1.

Type	Dilation	AF	Channels
3D Convolution	1	ReLU	8
3D Convolution + Max Pooling	1	ReLU	8
3D Convolution + Max Pooling	1	ReLU	4
3D Convolution + Max Pooling	1	ReLU	4
Dilated 3D Convolution	2	ReLU	4
Dilated 3D Convolution	2	ReLU	4
3D Convolution	1	ReLU	8
3D Convolution + Max Pooling	1	ReLU	8
3D Up-Sampling	1	ReLU	4
3D Up-Sampling	1	ReLU	8
3D Up-Sampling	1	ReLU	8
3D Up-Sampling	1	ReLU	8
3D Up-Sampling	1	ReLU	8
3D Convolution	1	Sigmoid	1

As illustrated in Figure 5, the 3D completion network output is followed by an up-sampling convolution layer and ReLU activations in the upper middle. The high-resolution defect model acts as the second input and undergoes a down-sampling convolution layer, followed by ReLU activations and max pooling operations. The two inputs are added up and go through an up-sampling convolution layer with ReLU activations, as depicted on the upper right side of Figure 5. Another decoder section follows, which encompasses an addition operation with the corresponding feature maps from the down-sampling section. The bottleneck of the shallower U-Net does not have dilated convolution.

The output is generated by a convolutional layer normalized to the range [0, 1] using sigmoid activation functions. Finally, the predicted voxel values are thresholded at 0.45 to transform the resultant models into binary values. The network has a total of 11,741 trainable parameters.

### 2.3 Deep-learning networks training

While training the 3D completion network and the resolution enhancement network, we utilized binary cross-entropy (Liu and Qi, 2017) as the loss function and Adadelta (Zeiler, 2012) as the optimizer. The binary cross-entropy (Liu and Qi, 2017) evaluates the proximity of the predicted probability of voxel values to the target values, where 1 or 0 represent the presence or absence of bone tissue, respectively. Adadelta (Zeiler, 2012) is an adaptive stochastic gradient descent algorithm that adjusts the learning rate without needing a parameter setting. All trainable parameters were randomly initialized (Skolnick et al., 2015).

We utilized 21,600 datasets consisting of 128 × 128 × 96 skull models to train the 3D completion network. In addition, we employed 5,800 datasets of 128 × 128 × 96 and 512 × 512 × 384 skull models to train the resolution enhancement network. During the network training phase, we used 2,400 datasets for the 3D completion network validation and 600 for the resolution enhancement network validation. These validation datasets were independent of the training datasets. All skull models were saved as uint8, where the file size of a 128 × 128 × 96 skull model was between 127 and 268 kB, and the file size of a 512 × 512 × 384 skull model was between 5.1 and 7.1 MB.

The calculations for network training and usage were performed on a personal computer that had an Intel Core i9-9900K 3.6 GHz CPU, 128 GB DDR4 memory, and an NVIDIA GeForce RTX A6000 graphics card with 48 GB GDDR6 GPU memory. To accommodate the GPU memory limitations, we used a batch size of 10 during the 3D completion network training and 4 during the resolution enhancement network training.

We shuffled the datasets at the beginning of each epoch to improve data order independence and prevent the optimizer from getting stuck in a local minimum of the loss function. The 3D completion network was trained for 1,200 epochs over 12.5 days, while the resolution enhancement network was trained for 20 epochs over 45 days. After training, we selected the networks that achieved the best loss values in the validation dataset for the reconstruction and resolution enhancement tasks.

Following the training, it took only 4.9 s to obtain a completed 128 × 128 × 96 skull model using the 3D completion network and 7.2 s to get a 512 × 512 × 384 high-resolution skull model using the resolution enhancement network. In summary, using the proposed approach, it takes less than 10 min to create an implant model ready for manufacturing once a defective skull model is available for design. This is a significant improvement over the manual restoration method, which takes more than 1 h. This is in addition to the improvement in geometric quality.

Further details regarding the hardware and software setup for training and evaluation, training history, and additional case studies are available in the Supplementary Material linked to this article.

## 3 Results

To demonstrate the performance of our proposed 3D cranial inpainting system, both numerical studies and surgical practice are presented in this section, highlighting its quantitative and qualitative capabilities.

### 3.1 Numerical study

We created defects on numerical models by applying various 3D masks to intact skull models for study. The removed parts are considered ground truth (ideal) implants for quantitative investigations. The implants generated by the proposed system are compared to the ground truth implant models using the Sørensen-Dice index (Dice, 1945; Carass et al., 2020) and Hausdorff Distance (Morain-Nicolier et al., 2007) metrics.

In this study, skull models were converted to a voxel-based representation. Each voxel value is treated as a Boolean, with 1 representing bone tissue and 0 representing otherwise. The Sørensen-Dice index (SDI) (Dice, 1945; Carass et al., 2020) is defined and calculated as follows:

$$SDI=\frac{2N_{TP}}{2N_{TP}+N_{FP}+N_{FN}}\times 100\;\%=\frac{2{\left\|P\cap G\right\|}_{1}}{{\left\|P\right\|}_{1}+{\left\|G\right\|}_{1}}\times 100\;\%$$ (1)

In Eq. 1, *N*_*TP*_ denotes the number of true positives, *N*_*FP*_ represents the number of false positives, and *N*_*FN*_ is the number of false negatives. In the second expression of Eq. 1, *P* is a skull model predicted by the proposed approach, and *G* is its corresponding ground-truth model. The 1-norm calculates the number of 1’s in a voxel-based model.

In addition, the Hausdorff Distance (HD) (Morain-Nicolier et al., 2007) measures the distance between two sets of voxels, *P* and *G,* in this study. It is defined as the most significant distance from the center of any bone-tissue voxel in the set *P* to the closest center of any bone-tissue voxel in the set *G*. The HD between *G* and *P* is calculated as:

$$\begin{array}{ccc} HD & = & \max_{g_{B}\in G\;}\min_{p_{B}\in P}{\left\|g_{B}-p_{B}\right\|}_{2} \end{array}$$ (2)

In Eq. 2, *g*_*B*_ and *p*_*B*_ represent bone-tissue voxels (with value 1) in *G* and *P*, respectively. The distance between the centers of two voxels is calculated using the *L* ^2^ norm (the Euclidean norm). For a skull model of voxels measuring 0.45 mm × 0.45 mm × 0.8 mm, one HD unit is equivalent to a distance between 0.45 mm and 1.0223 mm, serving as a quantitative measurement.

**FIGURE 6:**
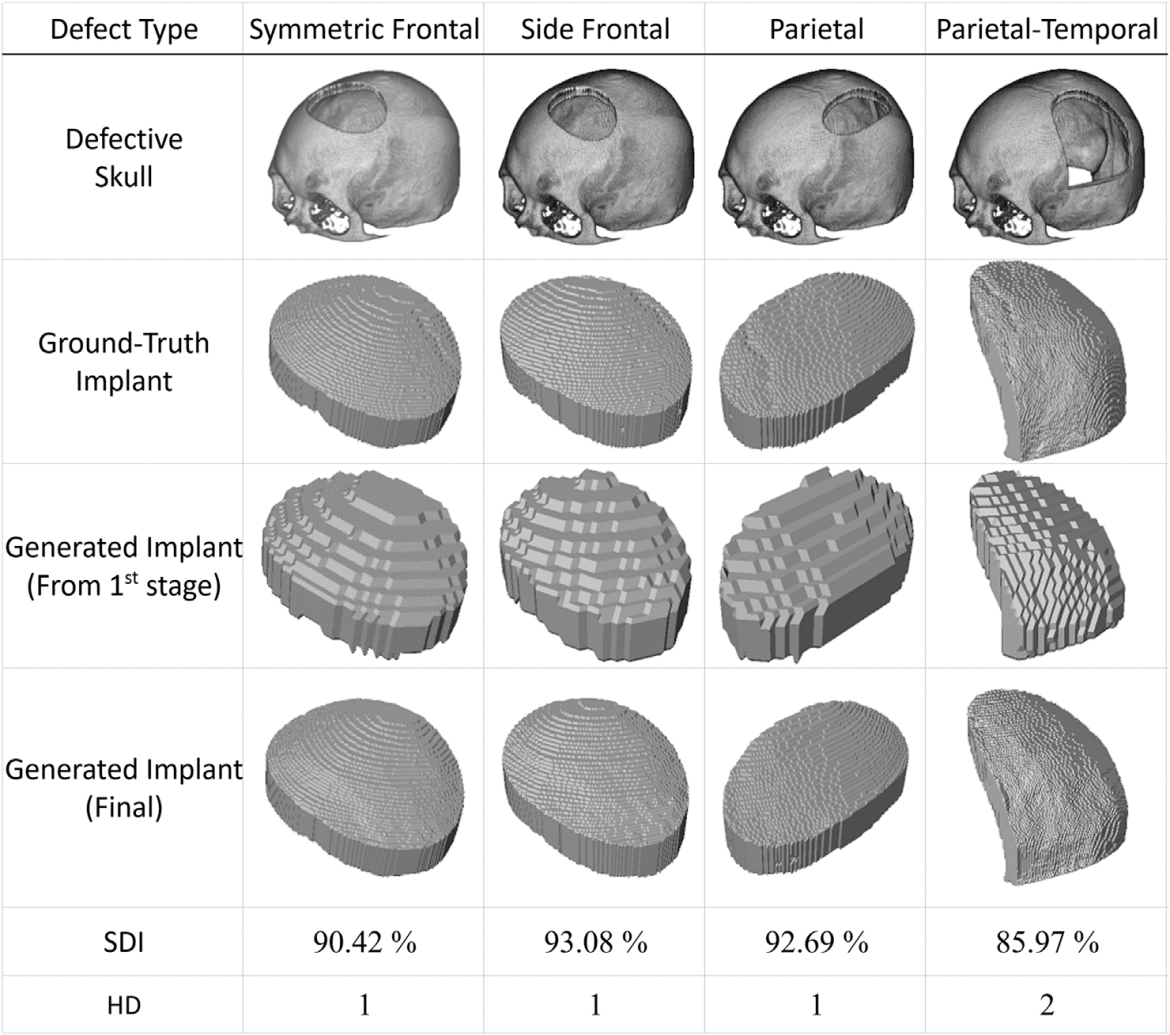
Automatic generation of implants for simulated defects using the proposed method. The first row shows skull models with simulated defects derived by subtracting 3D masks from an intact skull model using Boolean operations. The second row displays the ground-truth implants. The automatic generation of implants using the proposed approach requires two stages. The third row shows the outputs of the first stage, while the fourth row presents the results of the final stage. It is important to note that there is no cranial suture pattern on the resulting implant models and that the test datasets have never been used in training.. (All implant models, including the ground-truth implants, are enlarged to facilitate visual inspection).

The proposed approach generates implants in two stages, as detailed in the Materials and Methods section. The first stage produces low-resolution implant modes, while the second generates high-resolution ones. Figure 6 illustrates four case studies with simulated defects. The first row displays the defective skull models in an isometric view. The ground-truth implants are shown in the second row for comparison. The third and fourth rows present the low and high-resolution implants produced by the proposed system in the first and second stages, respectively.

The last two rows of Figure 6 present the quantitative performance of the proposed deep learning scheme. The case in the last column, with a significant defect denoted as type Parietal-Temporal, has an HD index of 2, while the rest have HD values of 1. The last column also has a smaller SDI value of 85.97%, while the SDI values for the remaining columns are all above 90%.

This simulation study shows that the suggested system can produce implants that closely resemble actual lost tissue based on defective skull models. The first stage generates implants with a lower resolution, while the second stage significantly enhances their resolution. Please note that cranial suture patterns are not restored, which does not hinder practical usage.

**FIGURE 7:**
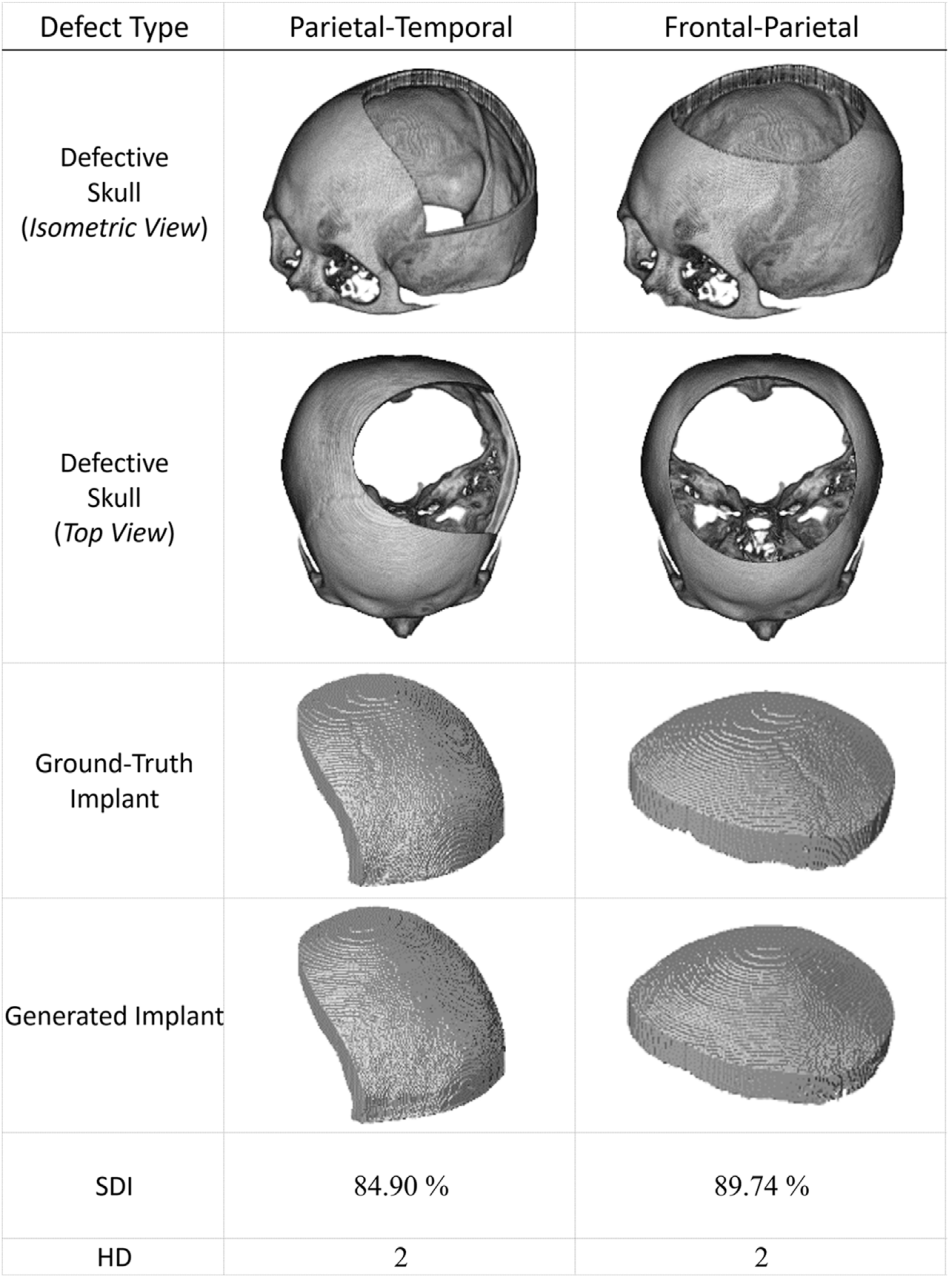
Two extreme cranial reconstruction examples with large-area defects. The first two rows display skull models from different viewpoints with defects more significant than one-third of the upper skull. The third row shows the ground-truth implant modes, while the fourth row presents the final implant models generated by the proposed scheme. (The ground-truth and generated implants have been magnified for visual inspection. Also, these two defective datasets have never been used in training).

Figure 7 presents two additional case studies demonstrating the proposed networks’ ability to reconstruct and improve the resolution of large-area defects. These two extreme cases illustrate the potential of the proposed system to reconstruct defects more significant than one-third of the upper part of the skull.

**FIGURE 8:**
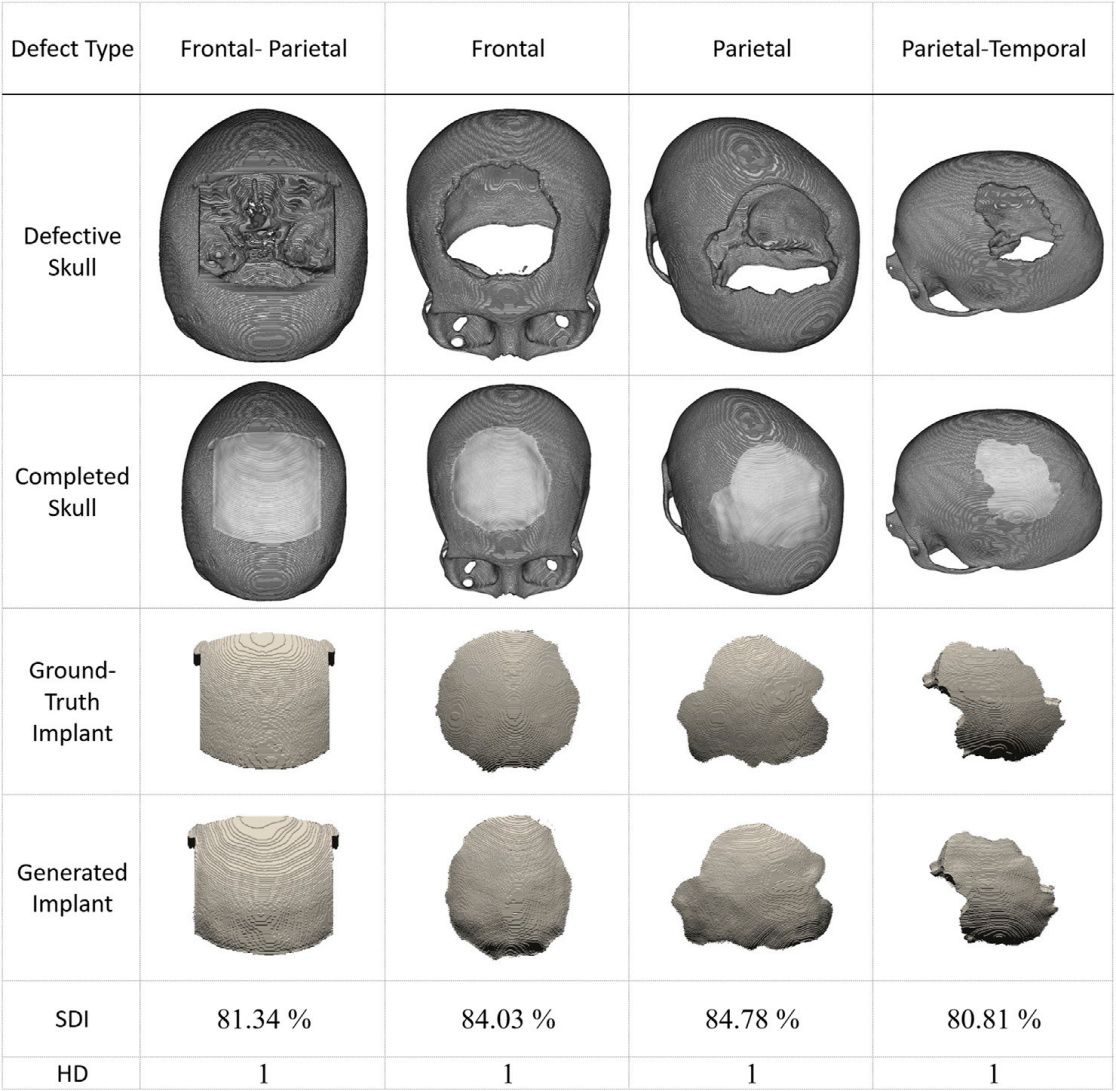
Inpainting performance of the proposed approach using cases collected from (Li and Egger, 2020). The first row shows defective skull models. The second row shows the generated implants placed on the defective models, demonstrating their compatibility with the defects. The third row displays the ground-truth implant modes, while the fourth row presents the final implant models generated by the proposed scheme.. (The implants are magnified in the third and fourth rows to facilitate visual inspection. These defective data sets have never been used in training).

Recently, several publicly available datasets have been released for cranial reconstruction studies. We collected several cases from (Li and Egger, 2020) and present four representative results in Figure 8. The defect in the first column is made of a cubic mask. In contrast, the defects in the other three columns are irregularly shaped. The first row displays the defective skulls, while the second row shows how the generated implants fit into them. The ground-truth and generated implants are shown in the third and fourth rows. The last two rows demonstrate the quantitative performance of the proposed system in these simulated cases.

Based on the results presented in Figure 8, we can conclude that the reconstruction performance of the proposed system degrades for irregular defects. However, HD values remain at 1, and all SDI values are above 80%.

Further analysis of the reconstruction performance of our approach is available in the Supplementary Material, which is linked to this article. These studies include comparisons with manually repaired cases stored in a database known as MUG500+ (Li et al., 2021c). One of the examples in the Supplementary Material, Supplementary Figure S11, demonstrates four cases with significant and irregular defects that were reconstructed using our method. The frontal-orbital implants have lower SDI values of 78.28% and 79.67% compared to the frontal-parietal implants, which have SDI values of 79.83% and 83.76%. This difference in performance may be due to the lack of frontal-orbital defective cases in the training dataset. However, implants created for these challenging cases are still useful for treatment purposes with minor modifications, even though the HD values go up to 2.

### 3.2 Surgical practice

The proposed deep learning system has been utilized in cranial surgeries, along with retrospective numerical studies presented in the last section. These studies have been registered on ClinicalTrials.gov with Protocol ID 202201082B0 and ClinicalTrials.gov ID NCT05603949. Additionally, the study has been approved by the Institutional Review Board of Chang Gung Medical Foundation in Taiwan under IRB 202002439B0. Here, we demonstrate a surgical application outcome of our proposed system.

**FIGURE 9:**
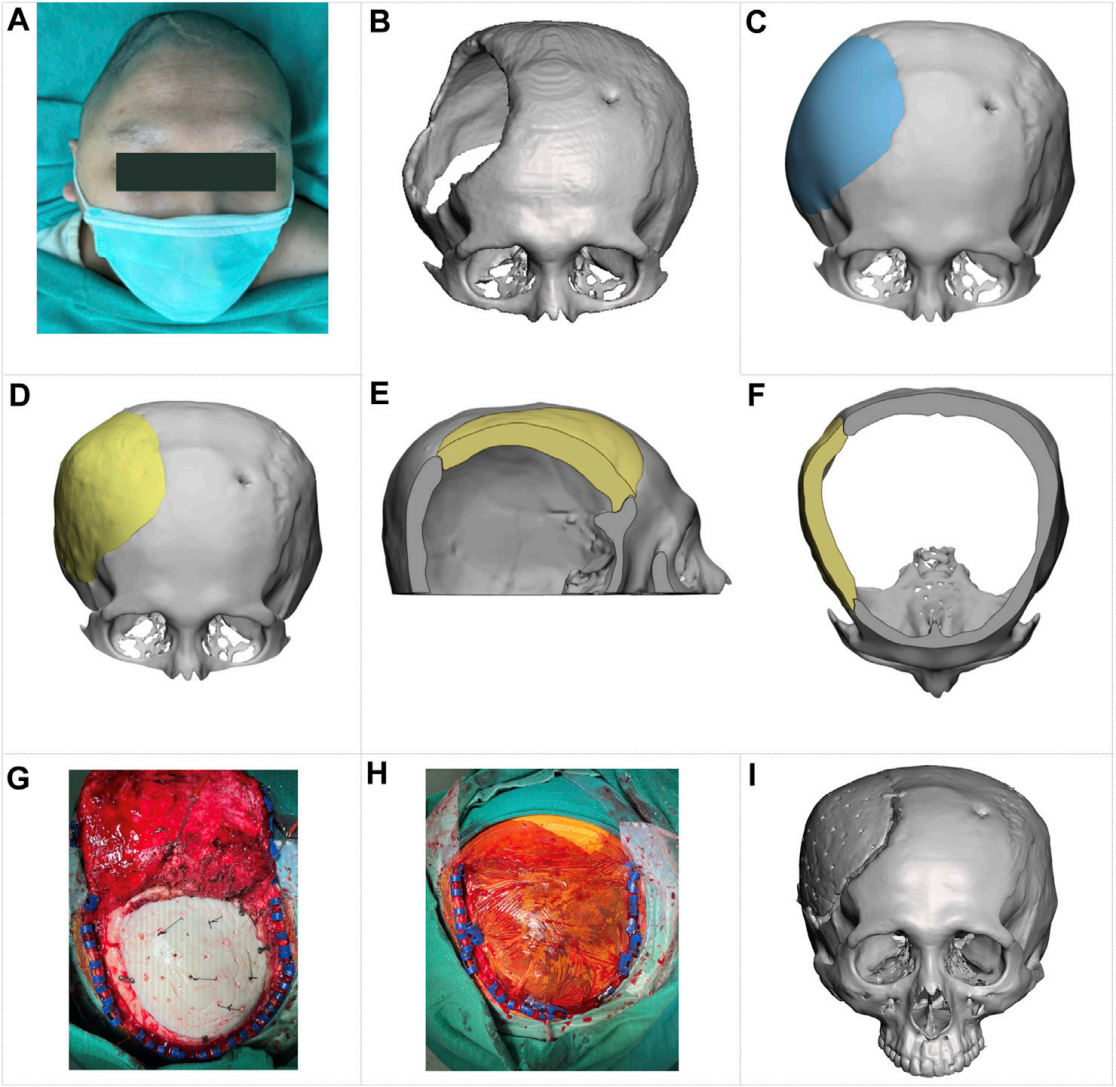
Clinical reconstruction of a skull defect with an implant designed by the proposed approach. **(A)** a preoperative photograph of the patient. **(B)** a 3D skull image based on a CT scan. **(C)** a skull model reconstructed by a technician using CAD software, showing the appearance of a typical hand-designed implant (depicted in blue for clarity). **(D–F)** illustrate the shape of the implant (in yellow) generated by the proposed approach and how it fits into the defective skull in the front view, sagittal cross-section view, and transversal cross-section view, respectively. **(G, H)** are intraoperative photographs. **(I)** a postoperative 3D image based on CT scanning, showing the patient’s cranial status 1 week after the surgery.

A young man, aged 24, has a significant craniofacial deformity and was seeking surgery to restore the structure of his skull. Seven years ago, the patient fell from a height of 5 m, resulting in a severely comminuted fracture and intracranial hemorrhage. These injuries were treated with an extensive craniectomy, but the skull has remained open, as shown in Figure 9A.

As depicted in the 3D image from a CT scan in Figure 9B, this cranial opening spanned the parietal, frontal, and temporal bones and measured up to 114 mm at its widest point. The edge of the opening was covered by scar tissue due to a prolonged ossification process. Additionally, there was a small hole in the left frontal bone to place an external ventricular drain.

Figures 4E, F, 9D show the shape of an implant generated by the proposed method and how it fits into the defective skull. For comparison, Figure 9C shows a reconstructed skull model created by a technician using CAD software to demonstrate what a typical hand-designed implant would look like. The implant produced by the proposed method fits the defect well and has a more natural appearance despite being asymmetrical to the left side of the skull. Additionally, a small patch covering the hole drilled for the ventriculostomy drainage system was removed, as placing an implant of that size was unnecessary.

To quantitatively assess the reconstruction performance, we compared the reconstructed 3D skull models generated by the proposed deep-learning approach with that designed manually using the cranial vault asymmetry index (CVAI) (Yin et al., 2015), originally used to evaluate the symmetry of positional plagiocephaly.

**FIGURE 10:**
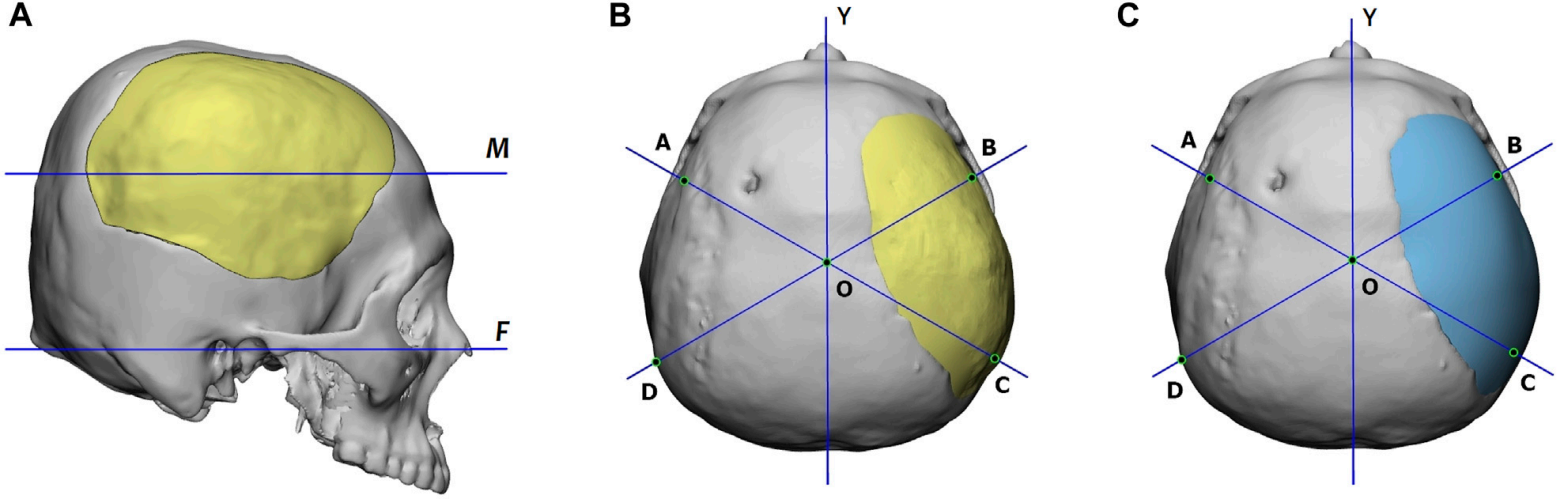
**(A)** Definition of the measurement plane, denoted as *M*, for the CVAI index. Plane *M* intersects the implant the most and is parallel to the Frankfort horizontal plane, marked as *F*. **(B)** and **(C)** are the reconstructed skulls’ top views, which display the diagonal lines for CVAI. Lines AC and BD are drawn 60° from the *Y*-axis in each diagram, and points A, B, C, and D are located on the measurement plane. The point o is located at the intersection of lines AC, BD, and the *Y*-axis. The implant of **(B)** is generated by the proposed deep-learning approach, while that of **(C)** is designed manually.

The CVAI index is calculated using a measurement plane. Figure 10 shows that this plane intersects the implant the most and is parallel to the Frankfort horizontal plane. Additionally, the figure provides top views of the reconstructed skulls, where lines AC and BD are diagonal lines drawn 60° from the *Y*-axis. Points A, B, C, and D are located on the measurement plane, and point O is at the intersection of lines AC, BD, and the *Y*-axis.

Based on the length of these lines, we define the anterior cranial vault asymmetry index (ACVAI) and the posterior cranial vault asymmetry index (PCVAI) in Eqs 3, 4.

$$ACVAI=\frac{\left|\left|\bar{AO}\right|-\left|\bar{BO}\right|\right|}{\left|\bar{AO}\right|}\times 100\;\%$$ (3)

$$PCVAI=\frac{\left|\left|\bar{CO}\right|-\left|\bar{DO}\right|\right|}{\left|\bar{DO}\right|}\times 100\;\%$$ (4)

The ACVAI evaluates the degree of asymmetry in the front part of the skull based on the intact side, while the PCVAI evaluates the back part of the skull. A perfectly symmetrical reconstructed skull will receive a score of 0% for both ACVAI and PCVAI.

Table 3 summarizes the ACVAI and PCVAI values of the two design approaches. The proposed deep-learning approach yielded ACVAI and PCVAI values of 2.22% and 2.14%, respectively. On the other hand, the manual design approach resulted in ACVAI and PCVAI values of 2.05% and 6.03%, respectively. The deep-learning approach produced more symmetric geometry in the back part of the skull, while the difference in the front part of the skull was insignificant for both approaches.

**TABLE 3:** Comparison of implant design quality using ACVAI (the anterior cranial vault asymmetry index) and PCVAI (the posterior cranial vault asymmetry index). Lengths of lines AO, BO, CO, and DO are measured in millimeters.

	AO	BO	CO	DO	ACVAI (%)	PCVAI (%)
Reconstruction method						
Deep-Learning	71.05	72.63	83.70	85.53	2.22	2.14
Manual		72.51	80.37		2.05	6.03

The implant fabrication process began with creating a 3D-printed template using the implant model generated by the proposed deep learning system. Silicone rubber was then used to make a mold that captured the geometric details of the implant. The implant was created through casting and molding, with most of the manufacturing in the operating room to ensure cleanliness and sterilization. In this surgery, the implant was made of polymethylmethacrylate (PMMA) (Yeap et al., 2019) bone cement. We have chosen this material for skull patches for over 15 years (Wu et al., 2021) and found it satisfactory in healing, duration, and providing protection. Excluding 3D printing, the casting and molding process took less than 30 min.

As shown in the intraoperative photograph in Figure 9G, we made 30 holes in the implant with a diameter of 2 mm for dural tenting (Przepiórka et al., 2019). In our experience with cranioplasty, this arrangement facilitates interstitial fluid circulation and exudate absorption during healing. Figure 9I shows a 3D image based on a CT scan taken 1 week after the surgery. The patient has been followed up for over 6 months and has no postoperative complications.

## 4 Discussion

According to (Li et al., 2021b), craniotomy defects typically have uneven borders due to manual cutting during the procedure. Our first-hand experience aligns with this observation. As a result, our study did not utilize synthetic defects with straight borders, as provided in, e.g., (Gall et al., 2019; Li et al., 2021d), to train and demonstrate the reconstruction capabilities of our proposed system.

As materials engineering advances, neurosurgeons explore using alloplastic materials (Yeap et al., 2019; Alkhaibary et al., 2020) for long-term skull reconstruction. Various options, such as polymethylmethacrylate (PMMA), polyetheretherketone (PEEK), polyethylene, titanium alloy, and calcium phosphate-based bone cement, have been used for cranioplasty materials. PEEK and titanium alloys offer excellent biomechanical properties, allowing for a significant reduction in implant thickness to reduce loading while providing support (PEEK’s tensile strength: 90 MPa; Ti6Al4V Grade 5: 862 MPa). To facilitate this, surgeons must be able to determine the thickness of an implant according to their requirements.

**FIGURE 11:**
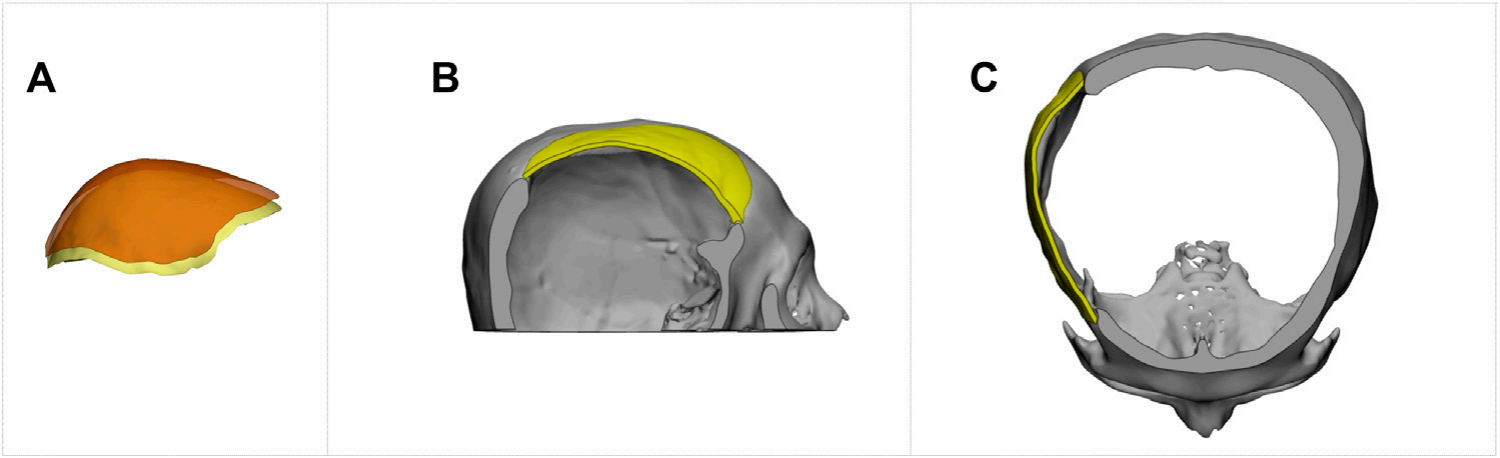
The creation of an implant with a specified thickness for cranial restoration. The implant model was generated by extracting the outer surface of the implant generated by the proposed method, shown in Figure 9D, and extending it by 2 mm in the inner direction. **(A)**. Surface extraction. **(B)**. Sagittal view. **(C)**. Transverse view.

To adjust the thickness of an implant model, one can utilize software tools like Autodesk^®^ Meshmixer to extract the outer surface. Extending the surface to a specified thickness, the final implant model can be produced. Figure 11 demonstrates this thickness-modification procedure using the defective cranial model described in Figure 9 with a 2 mm thickness for the new implant. Additionally, Ellis and coauthors in (Ellis et al., 2021) emphasized the importance of creating implants with smooth transitions and complete defect coverage without excess material. This example reveals that the updated design still fulfills these requirements, despite the change in thickness.

Our proposed system greatly minimizes the need for post-processing thanks to the remarkable similarity between the reconstructed and defective models. However, in line with the recommendation in (Li et al., 2021a), incorporating morphological openness and connected component analysis (CCA) (Gazagnes and Wilkinson, 2021) proves helpful in achieving the final implant geometry. Morphological openings, which involve erosion and dilation of the model, can eliminate small or thin noises attached to it and isolated noises. Also, verifying the final implant design using a 3D-printed model (Lee et al., 2009; He et al., 2016) before proceeding with cranioplasty is essential.

In clinical practice, allowing for larger tolerances when fitting a cranial implant may be necessary. One effective method to achieve this is to scale up the defective skull model to 102% before performing a Boolean subtraction with the reconstructed skull model. This approach provides a more tolerant fit and helps eliminate noise from mismatches between the reconstructed and defective models outside the defect area.

The training of the 3D completion network requires 1,200 epochs and 21,600 pairs of skull models, while that of the resolution enhancement network only needs 20 epochs and 5,800 pairs of skull models. This difference in data requirements and training epochs is due to the more significant challenge faced by the first network in reconstructing skulls with defects of varying sizes, positions, and types compared to that of the second network in raising the resolution of various skull models.

The proposed neural networks are based on the U-net (Ronneberger et al., 2015) architecture. The 3D completion network is a direct extension of the work presented in (Wu et al., 2022) and was constructed by increasing the resolution of each layer. The resolution enhancement network is created by merging two U-nets. This innovative architecture allows the network to effectively utilize the high-resolution geometry from the defective model and the low-resolution framework from the reconstructed model.

U-nets (Ronneberger et al., 2015) are autoencoders (Hinton and Salakhutdinov, 2006; Baldi, 2011; Dai et al., 2017) that feature skip connections (He et al., 2016). In a U-net, feature maps in the encoder section are combined with the corresponding feature maps in the decoder section. This makes the U-Net utilize features extracted in the encoder section to reconstruct a 3D model in the decoder part (Li et al., 2021a). showed the importance of skip connections (He et al., 2016) in encoder-decoder networks for reconstructing a defective skull.

We observed that the encoder-decoder network’s ability to fill holes decreased when skip connections were used. However, incorporating dilated convolutions (Yu and Koltun, 2016) compensates for this weakness and leads to stable convergence during training. This observation is consistent with the findings reported in (Jiang et al., 2020), which showed that dilation layers could enhance the performance of 2D image inpainting. Dilated convolutions allow kernels to expand their operating range on the input and gather contextual information from multiple scales. These features facilitate the completion of missing structures in the entire skull model.

Unlike the approach in (Devalla et al., 2018), which used dilated convolutions throughout, we applied dilated convolutions only to the bottleneck section in our proposed networks. We did not use batch normalization, as we did not observe accuracy benefits during network training, given memory and computing constraints limiting our batch size. We implement skip connections via summation (He et al., 2016) rather than concatenation (Gao Huang et al., 2017), as we found the summation operations more suitable for the voxel-based architecture to enable stable end-to-end training.

Through analysis of the training history and performance evaluations, we determined that removing batch normalization and restricting dilated convolutions simplifies the network, reduces computational overhead, and facilitates efficient training and inference while retaining accuracy. Specifically, attempting dilated convolutions throughout increased model capacity but resulted in instability during training and intractable execution time that hindered hyperparameter tuning. The arrangement of our architecture is tuned for the voxel input modality and tailored hardware constraints to enhance execution efficiency without sacrificing model performance.

In addition, the number of filters in the convolutional layers affects the stability and accuracy of the network. Increasing the filters can enhance capability but also prolongs costly 3D network training. Given our computational constraints, we optimized the filter numbers to balance performance and efficiency. Through experimentation, we found that 4-8 filters per layer provided adequate representational power while minimizing overhead.

While these choices are based on trial-and-error tuning, future ablation studies would provide a better understanding of each factor’s impact. Our current architecture modifications reduce parameters to facilitate efficient training under constraints. Further analysis can methodically validate the contribution of individual components like filter numbers to identify optimal accuracy-efficiency trade-offs based on available resources quantitatively.

## 5 Conclusion

A well-designed cranial implant improves aesthetic outcomes and minimizes operative duration, blood loss, and the risk of infection. This paper introduces an approach for automatically generating implant geometry using a deep learning system.

Our deep-learning approach’s success depends on two factors: the quality of the training data and the effectiveness of the neural network architectures. With our method, we can produce skull models with a volumetric resolution of 512 × 512 × 384 in two stages, which meets most clinical requirements for implant fabrication. In the first stage, the 3D completion network reconstructs defective skull models at a resolution of 128 × 128 × 96. In the second stage, another network known as the resolution enhancement network increases the reconstructed skull models’ resolution to 512 × 512 × 384.

Our numerical studies and clinical implementation have demonstrated the effectiveness of our proposed approach in creating personalized cranial implant designs for various clinical scenarios. The implants produced by the system were well-matched to the defects’ location and could significantly reduce surgery time. In a representative case study, our approach significantly produced more symmetric reconstruction than a manual design. This can lead to fewer postoperative complications and result in higher patient satisfaction.

Our research and clinical trials have demonstrated the effectiveness of our personalized cranial implant designs, which are tailored to different clinical scenarios. The implants generated by our system were accurately matched to the location of the defects, thereby significantly reducing surgery time. In a case study, our proposed approach produced more symmetric reconstructed geometry than manual design, leading to fewer postoperative complications and higher patient satisfaction.

This paper showcases the effectiveness of our proposed deep learning system for neurosurgery. However, we do acknowledge that our system has limitations. The process must be divided into two stages to reduce computational overhead during training, and the input needs to be normalized into two files with volume resolutions of 128 × 128 × 96 and 512 × 512 × 384. These limitations increase the amount of labor required and limit the range of deficiencies that can be addressed. In cases where defective skull models include even lower parts, designing implants using deep learning techniques may be more challenging or impossible due to individual differences in Zygomatic and Maxilla geometry.

We plan to further our research by developing a user-friendly system that is more computationally efficient, can recover a broader range of defect types and extents, and can accept datasets with varying patient poses and different slice intervals. To achieve these goals, we are exploring alternative deep learning networks based on other representations of 3D shapes, including polygon meshes (Hanocka et al., 2019), point clouds (Charles et al., 2017; Qi et al., 2017; Xie et al., 2021), and octree-based data (Tatarchenko et al., 2017; Wang et al., 2017; Wang et al., 2018; Wang et al., 2020). This could lead to more advanced and effective solutions than the volumetric data types used in our current work.

## Data Availability

The raw data supporting the conclusion of this article will be made available by the authors, without undue reservation.
