# Supplementary material for: Creating high-resolution 3D cranial implant geometry using deep learning techniques
Source: Front Bioeng Biotechnol. 2023 Dec 11;11:1297933. doi: 10.3389/fbioe.2023.1297933 (PMC10750412; doi:10.3389/fbioe.2023.1297933)
Supplement: Supplementary file 1 [file DataSheet1.PDF]

Supplementary Material

Creating High-Resolution 3D Cranial Implant Geometry Using Deep Learning Techniques

Chieh-Tsai Wu, Yao-Hung Yang, Yau-Zen Chang*

*** Correspondence:** Yau-Zen Chang: zen@cgu.edu.tw

This document provides the article's implementation details and demonstrates more performance evaluations. We used the same setup to train, evaluate, and utilize the proposed deep-learning networks.

The detailed architectures of the 3D completion network and the resolution enhancement network are shown in Supplementary Figure 1, Supplementary Figure 2, Supplementary Figure 3, and Supplementary Figure 4. When compiling these two networks, we used Adadelta as the loss function and binary cross-entropy as the optimizer.

All skull models were saved as uint8, the file size of a 128×128×96 skull model was between 127 and 268 kB, and the file size of a 512×512×384 skull model was between 5.1 and 7.1 MB. Due to the memory limitations of the graphics card, the batch size was set to 10 in training the 3D completion network and 4 in training the resolution augmentation network.

The training records of these two networks are shown in Supplementary Figure 5 and Supplementary Figure 6. When training the networks, 10 % of the dataset is used for validation, and the validation data is not used for training. It took 12.5 days to train the 3D completion network for 1,200 epochs using 24,000 pairs of 128×128×96 skull models. For the resolution enhancement network, it took 45 days to train for 20 epochs using 5,800 sets of 128×128×96 and 512×512×384 skull model combinations, which were randomly selected from the 24,000 pairs of the collected skull dataset. The test dataset was independent of the training dataset and the validation dataset.

After training, it took 4.9 seconds to obtain a completed 128×128×96 skull model using the 3D completion network and 7.2 seconds to get a 512×512×384 high-resolution skull model using the resolution enhancement network. Additional time is required for data processing, however. Loading the skull models into memory might take up to 30 minutes. The implant models were transformed into binary voxel values by thresholding voxel values at 0.45.

The overall inpainting performance of the proposed method on the Hausdorff distance (HD) and Sørensen-Dice index (SDI) distributions for the validation and test datasets are shown in Supplementary Figure 7 and Supplementary Figure 8, respectively. HD and SDI values were calculated between the generated and ground-truth implants.

Supplementary Figure 9 shows a skull reconstruction example with a parietotemporal defect more significant than two-fifths of the upper skull. This example

demonstrates a failed case using the proposed method. As shown in the isometric view of the reconstructed skull, the implant could not provide a smooth transition and cover the entire defect area. Nevertheless, the generated implant was close to the ground truth, which provided a valuable basis for the manual design using CAD software to create an applicable implant geometry.

The first row of Supplementary Figure 10 shows an isometric image when an implant generated by the proposed method is placed in a defective skull (the implant is depicted in blue for clarity). The second, third, and fourth rows show transverse, sagittal, and coronal cross-sectional views to facilitate implant thickness and matching border inspection.

Supplementary Figure 11 shows more reconstruction examples created by the proposed method. It can be seen that the SDI values of the frontal-orbital implants are worse than those of the frontal-parietal implants. This may be because we did not include any frontal-orbital defective examples in the training dataset.

Supplementary Figure 12 compares the implant generation performance of the proposed approach with the manual restoration method using models and implants provided in a database called the MUG500+ database. The cranial implants supplied in the database were designed manually by an expert. Since most problems in the database are contaminated, oversized, and irregular defective skull models beyond the repair capability of the proposed method, only four case studies are presented. Supplementary Figure 13 shows more diagrams and a photo of the clinical reconstruction case presented in the article.

Visualization of learnable parameters and feature maps provides a deeper understanding of deep learning systems. As shown in Supplementary Figure 14 to S20, we can visualize the kernels of the deep learning systems as 3×3×3 cubes. Dark voxels represent smaller weights, while light-colored squares represent larger weights.

It is worth noting that the feature maps in Supplementary Figure 15 and S20 are implant models. This is to be expected since the outputs of the two networks are the repaired skull models. Ideally, each output combines a defective model and an implant. The proposed networks can directly provide implant models by tapping the internal feature maps.

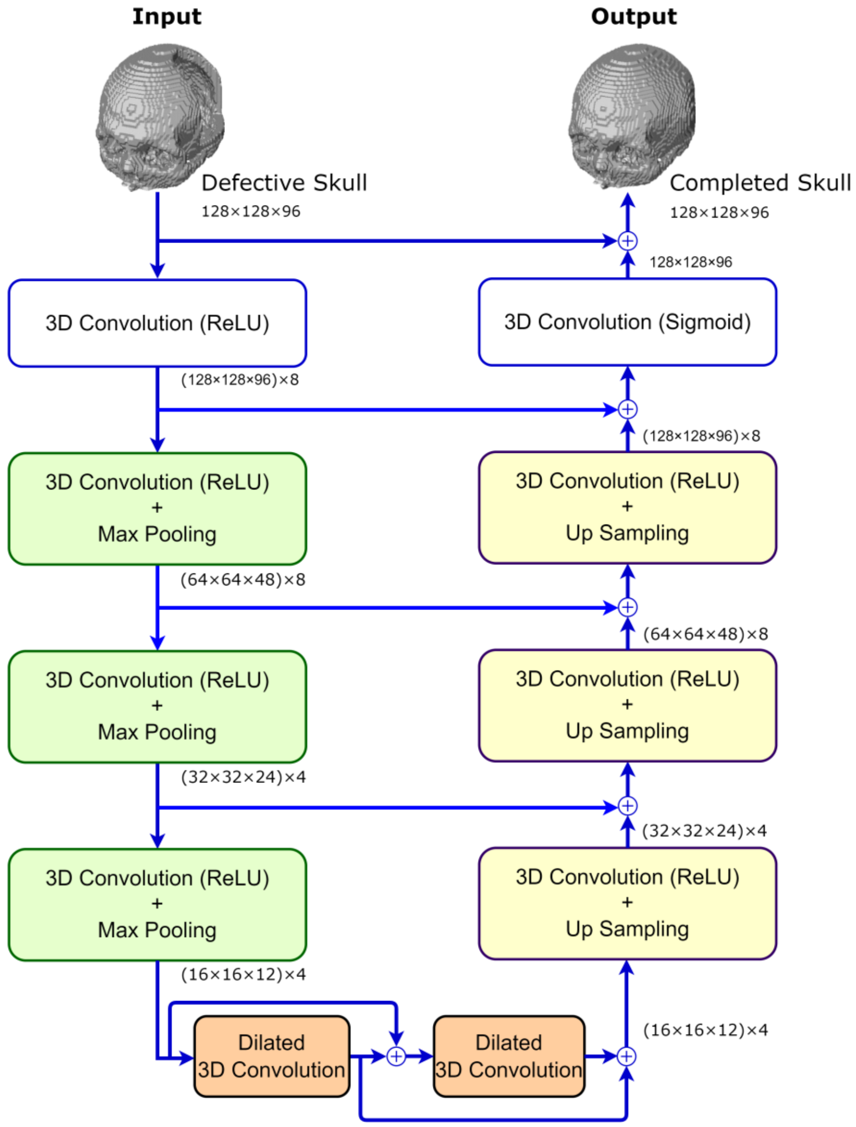

Supplementary Figure 1. The architecture diagram of the 3D completion network.

Layer (type)	Output Shape	Param #	Connected to
input_1 (InputLayer)	(None, 128, 128, 96)	0	
conv3d_1 (Conv3D)	(None, 128, 128, 96)	224	input_1[0][0]
conv3d_2 (Conv3D)	(None, 128, 128, 96)	1736	conv3d_1[0][0]
max_pooling3d_1 (MaxPooling3D)	(None, 64, 64, 48, 8)	0	conv3d_2[0][0]
conv3d_3 (Conv3D)	(None, 64, 64, 48, 4)	868	max_pooling3d_1[0][0]
max_pooling3d_2 (MaxPooling3D)	(None, 32, 32, 24, 4)	0	conv3d_3[0][0]
conv3d_4 (Conv3D)	(None, 32, 32, 24, 4)	436	max_pooling3d_2[0][0]
max_pooling3d_3 (MaxPooling3D)	(None, 16, 16, 12, 4)	0	conv3d_4[0][0]
conv3d_5 (Conv3D)	(None, 16, 16, 12, 4)	436	max_pooling3d_3[0][0]
add_1 (Add)	(None, 16, 16, 12, 4)	0	max_pooling3d_3[0][0]<br>conv3d_5[0][0]
conv3d_6 (Conv3D)	(None, 16, 16, 12, 4)	436	add_1[0][0]
add_2 (Add)	(None, 16, 16, 12, 4)	0	conv3d_5[0][0]<br>conv3d_6[0][0]
conv3d_7 (Conv3D)	(None, 16, 16, 12, 4)	436	add_2[0][0]
add_3 (Add)	(None, 16, 16, 12, 4)	0	conv3d_6[0][0]<br>conv3d_7[0][0]
cconv3d_8 (Conv3D)	(None, 16, 16, 12, 4)	436	add_2[0][0]
add_4 (Add)	(None, 16, 16, 12, 4)	0	conv3d_7[0][0]<br>conv3d_8[0][0]

conv3d_9 (Conv3D)	(None, 16, 16, 12, 4)	436	add_4[0][0]
up_sampling3d_1 (UpSampling3D)	(None, 32, 32, 24, 4)	0	conv3d_9[0][0]
add_5 (Add)	(None, 32, 32, 24, 4)	0	max_pooling3d_2[0][0]<br>up_sampling3d_1[0][0]
conv3d_10 (Conv3D)	(None, 32, 32, 24, 4)	872	add_5[0][0]
up_sampling3d_2 (UpSampling3D)	(None, 64, 64, 48, 8)	0	conv3d_10[0][0]
add_6 (Add)	(None, 64, 64, 48, 8)	0	max_pooling3d_1[0][0]<br>up_sampling3d_2[0][0]
conv3d_11 (Conv3D)	(None, 64, 64, 48, 8)	1,736	add_6[0][0]
up_sampling3d_3 (UpSampling3D)	(None, 128, 128, 96, 8)	0	conv3d_11[0][0]
add_7 (Add)	(None, 128, 128, 96, 8)	0	conv3d_1[0][0]<br>up_sampling3d_3[0][0]
conv3d_12 (Conv3D)	(None, 128, 128, 96, 1)	217	add_7[0][0]
add_8 (Add)	(None, 128, 128, 96, 1)	0	input_1[0][0]<br>conv3d_12[0][0]

Total params: 8,269

Trainable params: 8,269

Non-trainable params: 0

Optimizer: adadelta

Loss: binary_crossentropy

Supplementary Figure 2. A summary of the architecture of the 3D Completion Network. (Generated by the summary() function of Keras.)

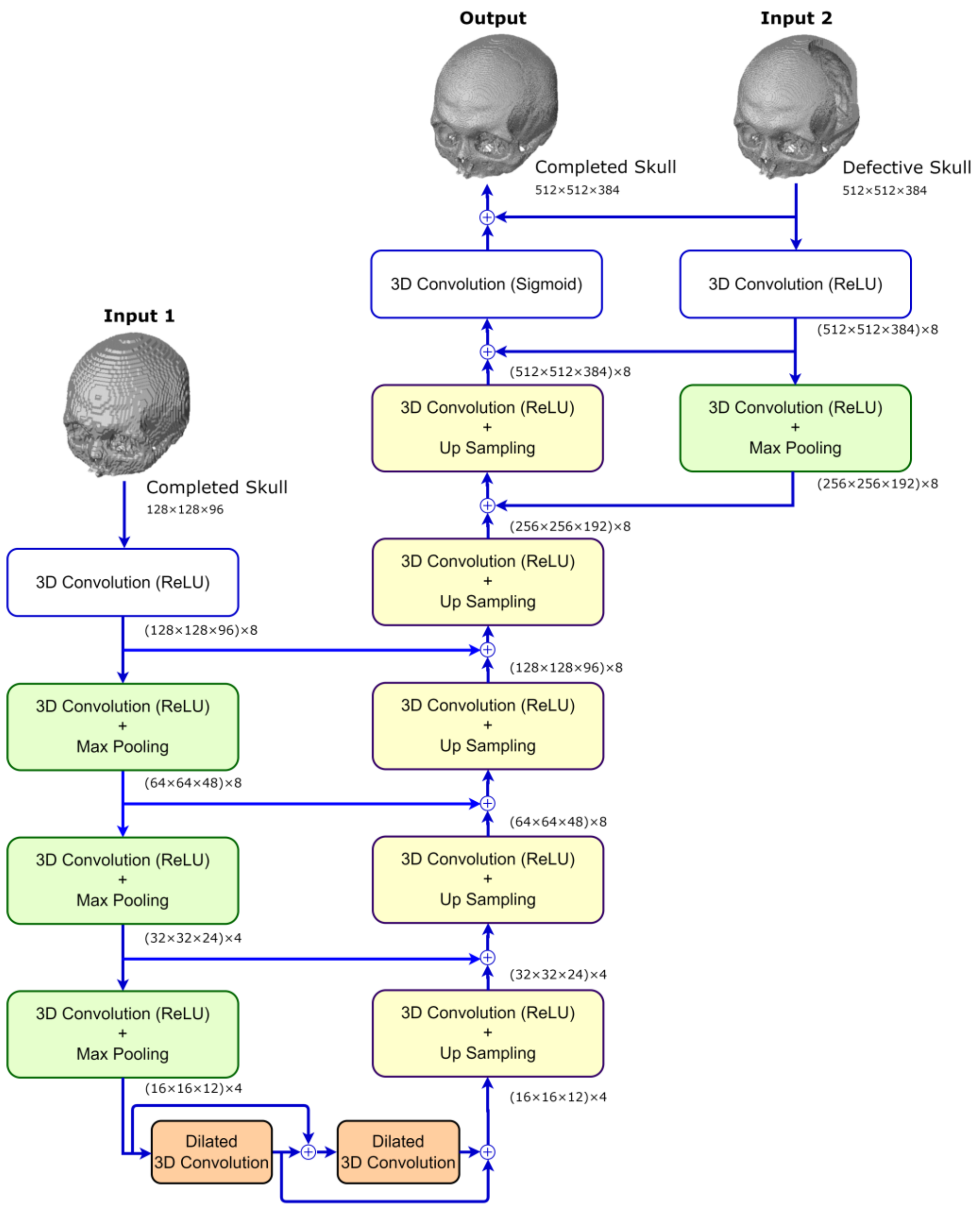

Supplementary Figure 3. The architecture diagram of the Resolution Enhancement Network.

Layer (type)	Output Shape	Param #	Connected to
input_1 (InputLayer)	(None, 128, 128, 96)	0	
conv3d_1 (Conv3D)	(None, 128, 128, 96)	224	input_1[0][0]
conv3d_2 (Conv3D)	(None, 128, 128, 96)	1736	conv3d_1[0][0]
max_pooling3d_1(MaxPooling3D)	(None, 64, 64, 48, 8)	0	conv3d_2[0][0]
conv3d_3 (Conv3D)	(None, 64, 64, 48, 4)	868	max_pooling3d_1[0][0]
max_pooling3d_2 (MaxPooling3D)	(None, 32, 32, 24, 4)	0	conv3d_3[0][0]
conv3d_4 (Conv3D)	(None, 32, 32, 24, 4)	436	max_pooling3d_2[0][0]
max_pooling3d_3 (MaxPooling3D)	(None, 16, 16, 12, 4)	0	conv3d_4[0][0]
conv3d_5 (Conv3D)	(None, 16, 16, 12, 4)	436	max_pooling3d_3[0][0]
add_1 (Add)	(None, 16, 16, 12, 4)	0	max_pooling3d_3[0][0]<br>conv3d_5[0][0]
conv3d_6 (Conv3D)	(None, 16, 16, 12, 4)	436	add_1[0][0]
add_2 (Add)	(None, 16, 16, 12, 4)	0	conv3d_5[0][0]<br>conv3d_6[0][0]
conv3d_7 (Conv3D)	(None, 16, 16, 12, 4)	436	add_2[0][0]
add_3 (Add)	(None, 16, 16, 12, 4)	0	conv3d_6[0][0]<br>conv3d_7[0][0]

cconv3d_8 (Conv3D)	(None, 16, 16, 12, 4)	436	add_2[0][0]
add_4 (Add)	(None, 16, 16, 12, 4)	0	conv3d_7[0][0]<br>conv3d_8[0][0]
conv3d_9 (Conv3D)	(None, 16, 16, 12, 4)	436	add_4[0][0]
up_sampling3d_1 (UpSampling3D)	(None, 32, 32, 24, 4)	0	conv3d_9[0][0]
add_5 (Add)	(None, 32, 32, 24, 4)	0	max_pooling3d_2[0][0]<br>up_sampling3d_1[0][0]
conv3d_10 (Conv3D)	(None, 32, 32, 24, 8)	872	add_5[0][0]
up_sampling3d_2 (UpSampling3D)	(None, 64, 64, 48, 8)	0	conv3d_10[0][0]
add_6 (Add)	(None, 64, 64, 48, 8)	0	max_pooling3d_1[0][0]<br>up_sampling3d_2[0][0]
conv3d_11 (Conv3D)	(None, 64, 64, 48, 8)	1,736	add_6[0][0]
input_2 (InputLayer)	(None, 512, 512, 384)	0	
up_sampling3d_3 (UpSampling3D)	(None, 128, 128, 96, 8)	0	conv3d_11[0][0]
conv3d_1 (Conv3D)	(None, 512, 512, 384)	1736	conv3d_1[0][0]
add_7 (Add)	(None, 128, 128, 96)	0	conv3d_3[0][0]<br>up_sampling3d_2[0][0]
conv3d_12 (Conv3D)	(None, 128, 128, 96)	1736	add_7[0][0]
max_pooling3d_1 (MaxPooling3D)	(None, 256, 256, 192)	0	conv3d_2[0][0]
up_sampling3d_4 (UpSampling3D)	(None, 256, 256, 192)	0	conv3d_12[0][0]
add_8 (Add)	(None, 256, 256, 192)	0	max_pooling3d_1 [0][0]<br>up_sampling3d_4[0][0]

conv3d_13 (Conv3D)	(None, 256, 256, 192)	1736	add_8 [0][0]
up_sampling3d_5 (UpSampling3D)	(None, 512, 512, 384)	0	conv3d_13[0][0]
add_9 (Add)	(None, 512, 512, 384)	0	conv3d_13 [0][0]<br>up_sampling3d_5[0][0]
conv3d_14 (Conv3D)	(None, 512, 512, 384,)	217	add_9[0][0]
add_10 (Add)	(None, 512, 512, 384)	0	input_2[0][0]<br>conv3d_14[0][0]

Total params: 11,741

Trainable params: 11,741

Non-trainable params: 0

Optimizer: adadelta

Loss: binary_crossentropy

Supplementary Figure 4. A summary of the architecture of the Resolution Enhancement Network. (Generated by the summary() function of Keras.)

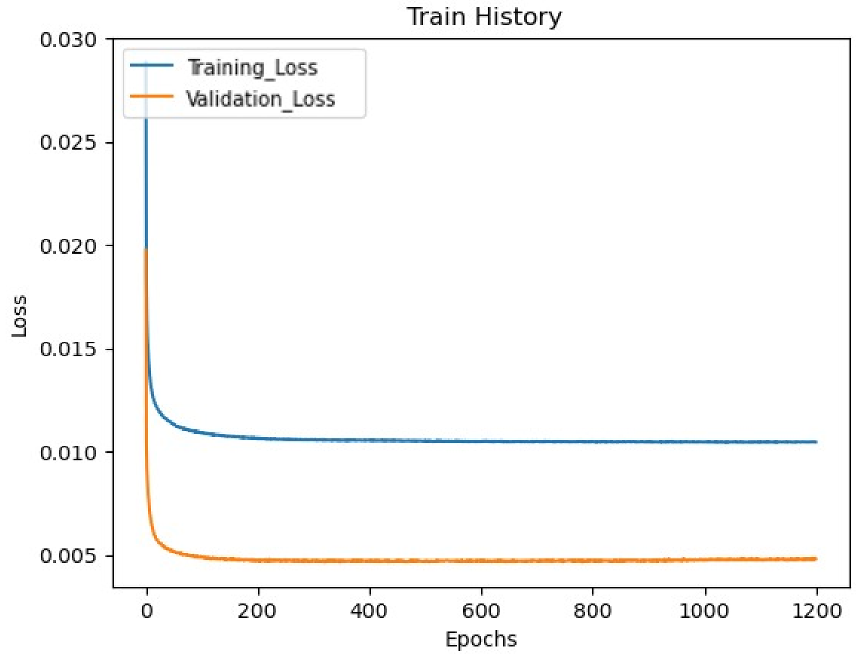

Supplementary Figure 5. Training history of the 3D Completion Network. There were 1,200 epochs overall.

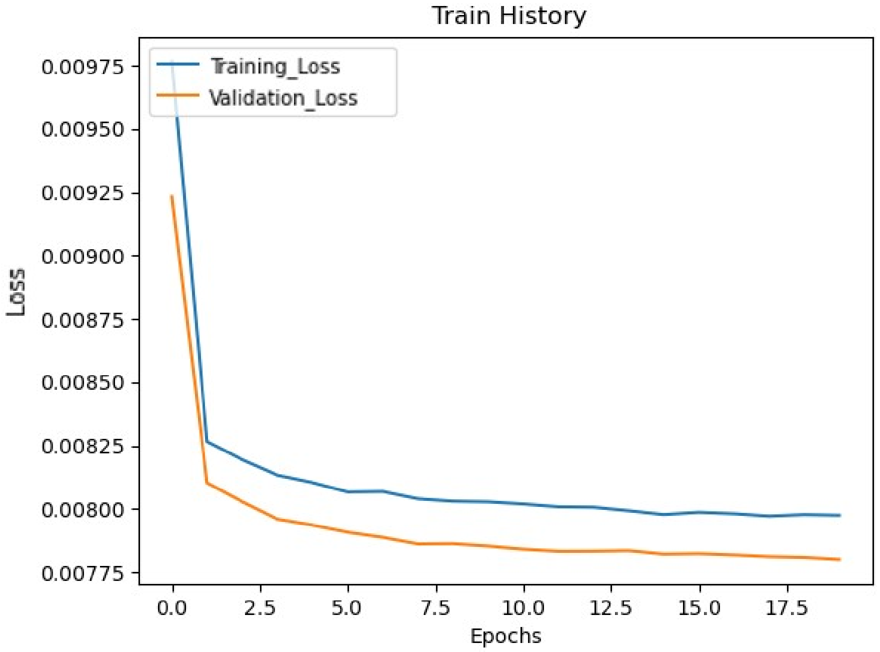

Supplementary Figure 6. Training history of the Resolution Enhancement Network. There were 20 epochs overall.

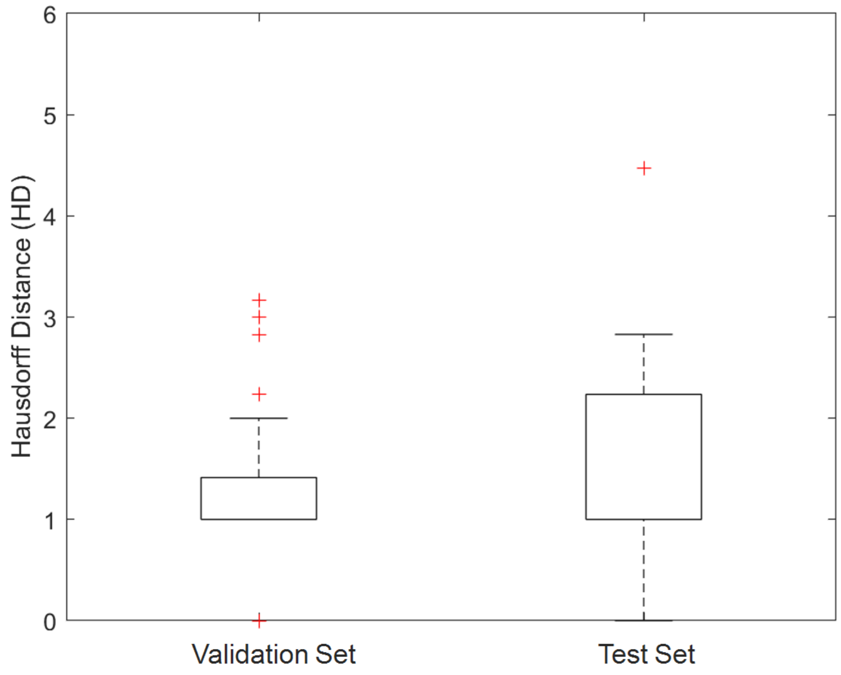

Supplementary Figure 7. Overall HD performance of the proposed approach on the validation and test datasets. The HD values were calculated between the generated implants and the ground-truth implants.

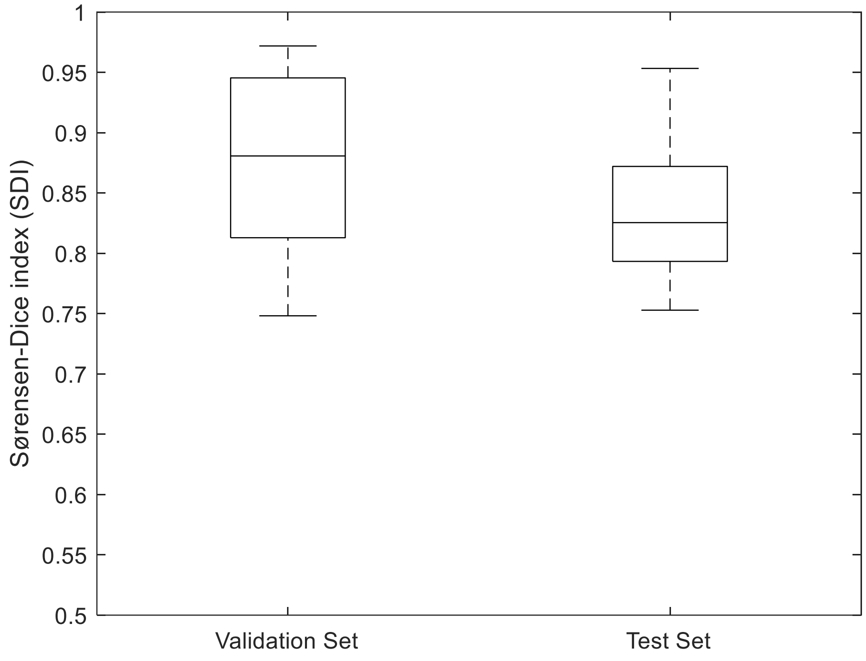

Supplementary Figure 8. Overall SDI performance of the proposed approach on the validation and test datasets. The SDI values were calculated between the generated implants and the ground-truth implants.

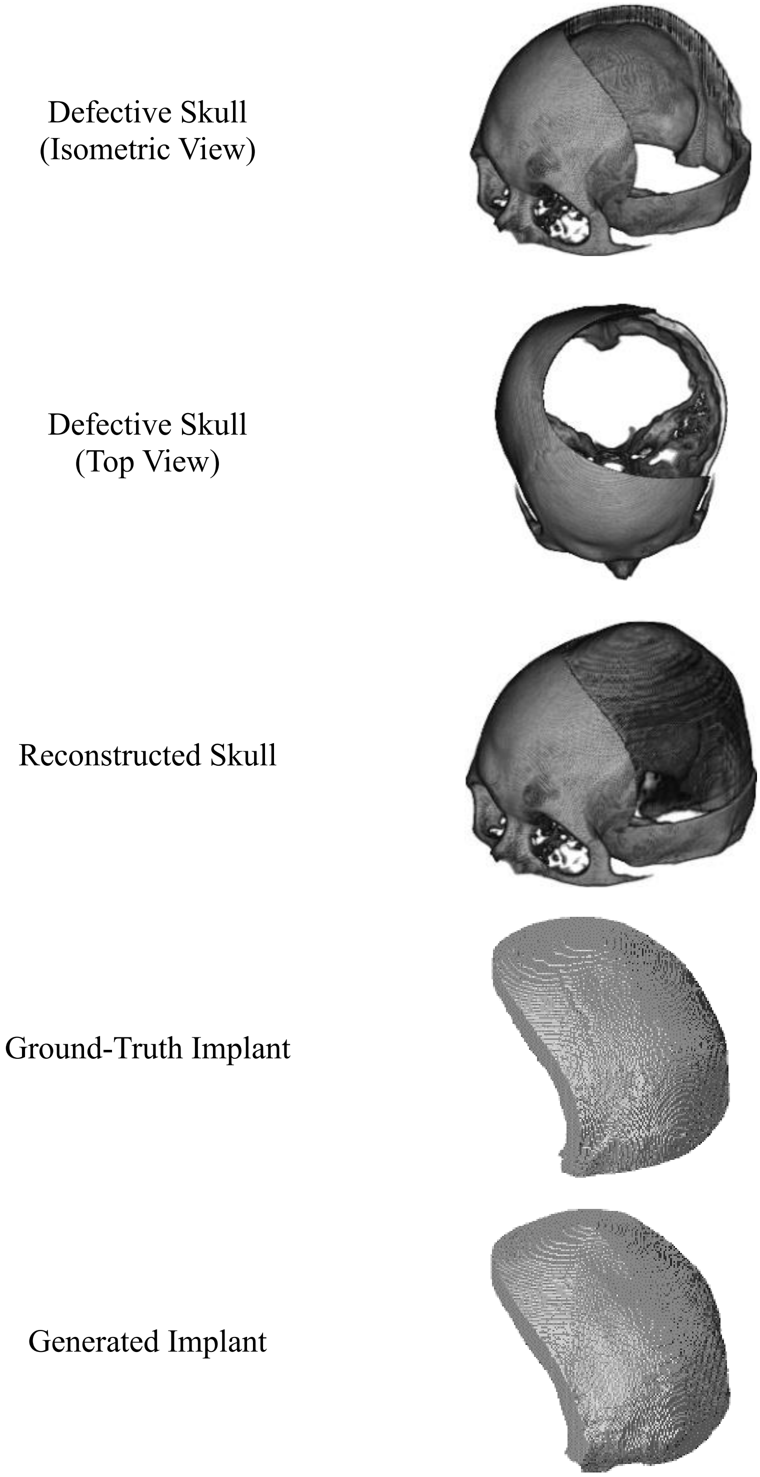

Supplementary Figure 9. Cranial reconstruction with a large-area defect. In this failed case, the parietotemporal defect is more significant than two-fifths of the upper skull. The isometric view of the reconstructed skull shows that the implant cannot cover the entire defect area. This can be observed in the lower part of the generated implant, which is significantly different from the ground-truth implant.

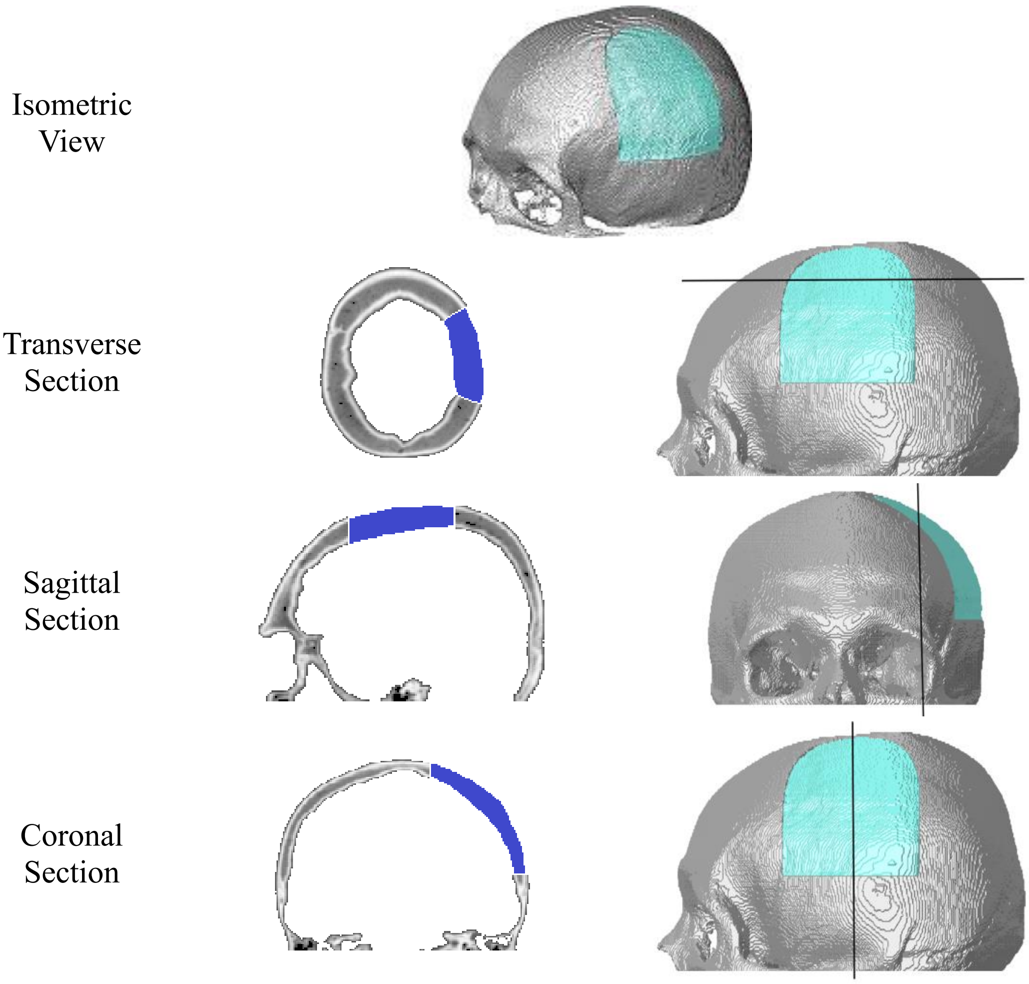

Supplementary Figure 10. Cross-sectional views of a parietal-temporal defect reconstructed by the proposed method. These images show the implant placed in the defective skull (implants are depicted in blue for clarity). The second, third, and fourth rows show lateral, sagittal, and coronal cross-sectional views.

Reconstructing Irregular Defects by the Proposed Scheme

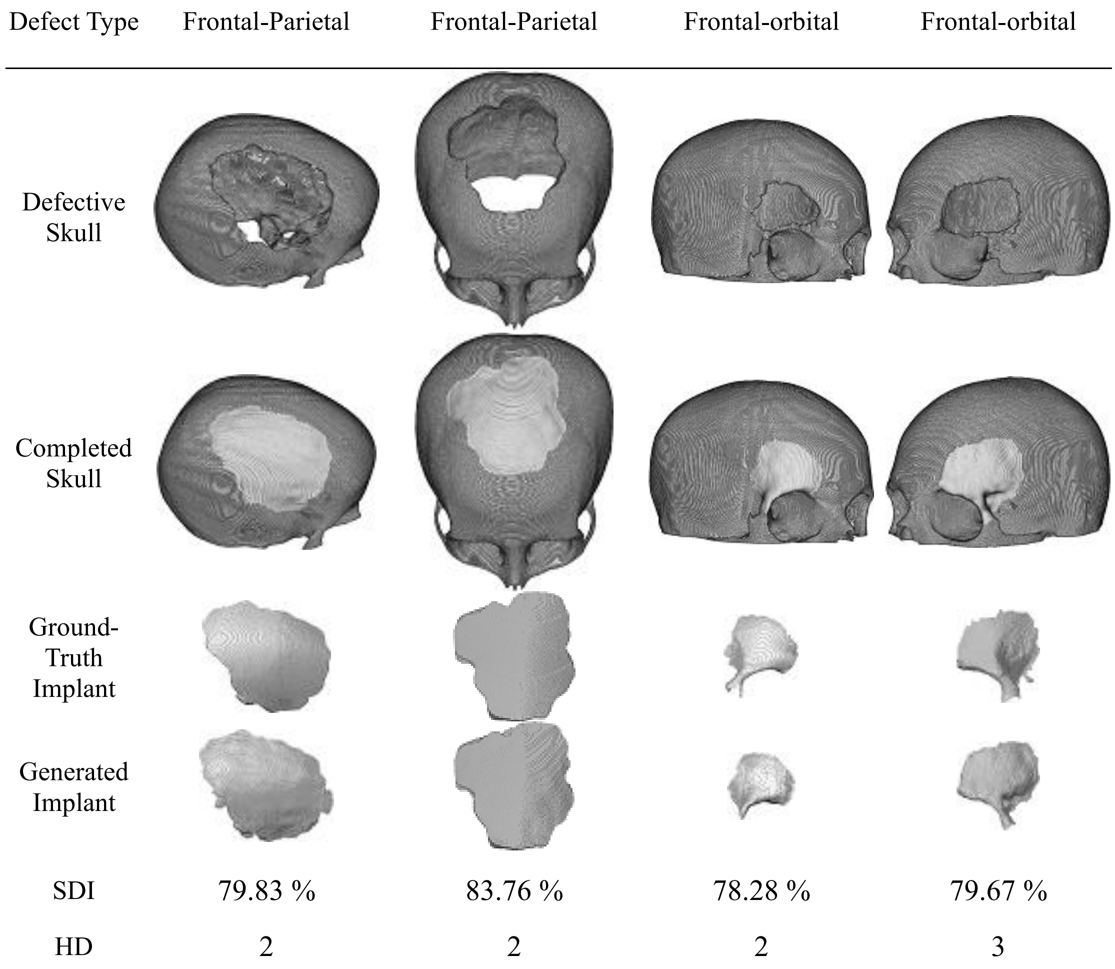

Supplementary Figure 11. More reconstruction examples created by the proposed approach.

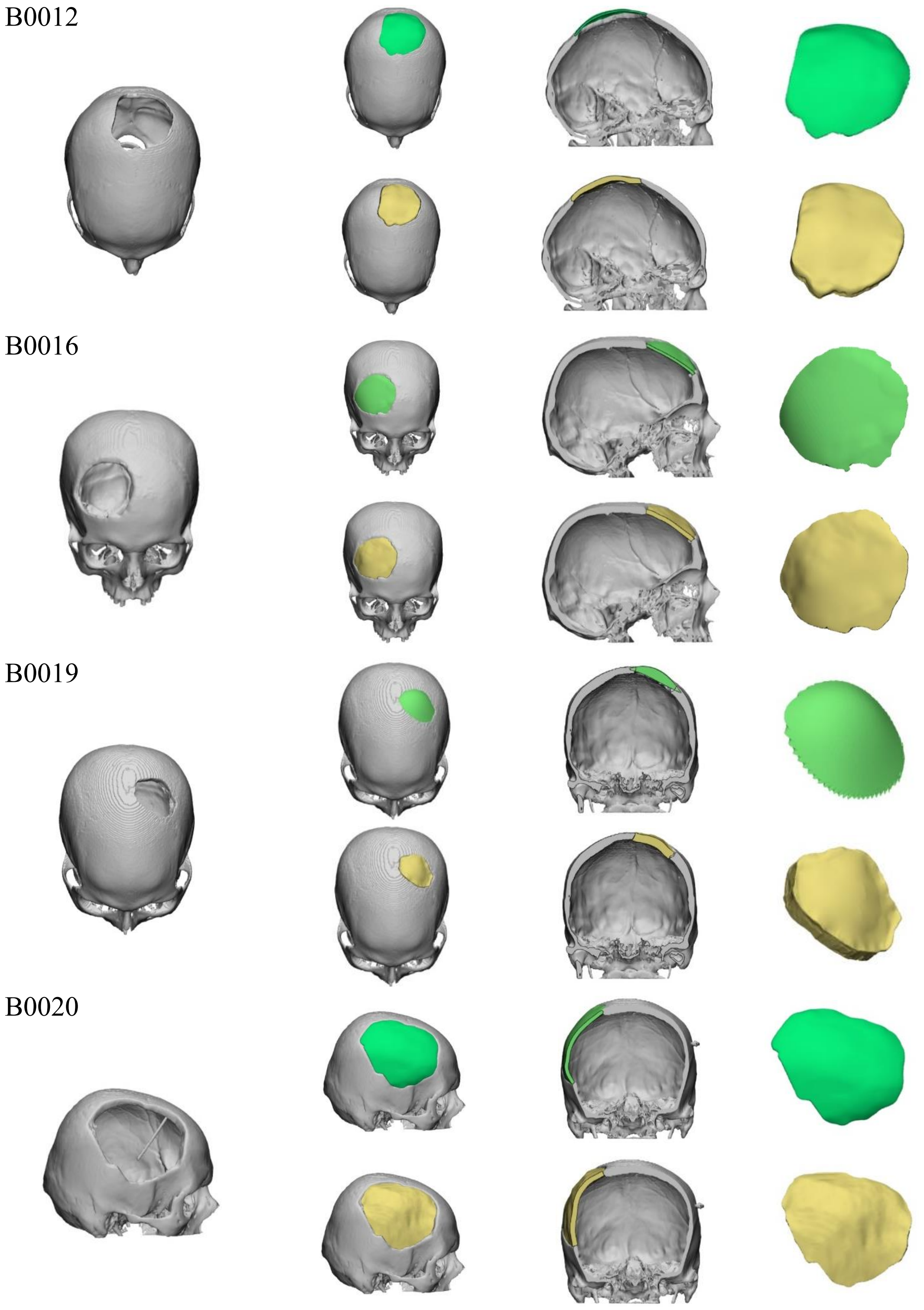

Supplementary Figure 12. Comparisons in the implant generation performance between the proposed approach and the manual restoration method. The four defective skulls, including the manually designed cranial implants (shown in green), were provided by the MUG500+ database (cited as ref. 33 in the article). Implants produced by the proposed method are shown in yellow. All implant pictures are magnified for easy inspection. Original serial numbers in the database are included.

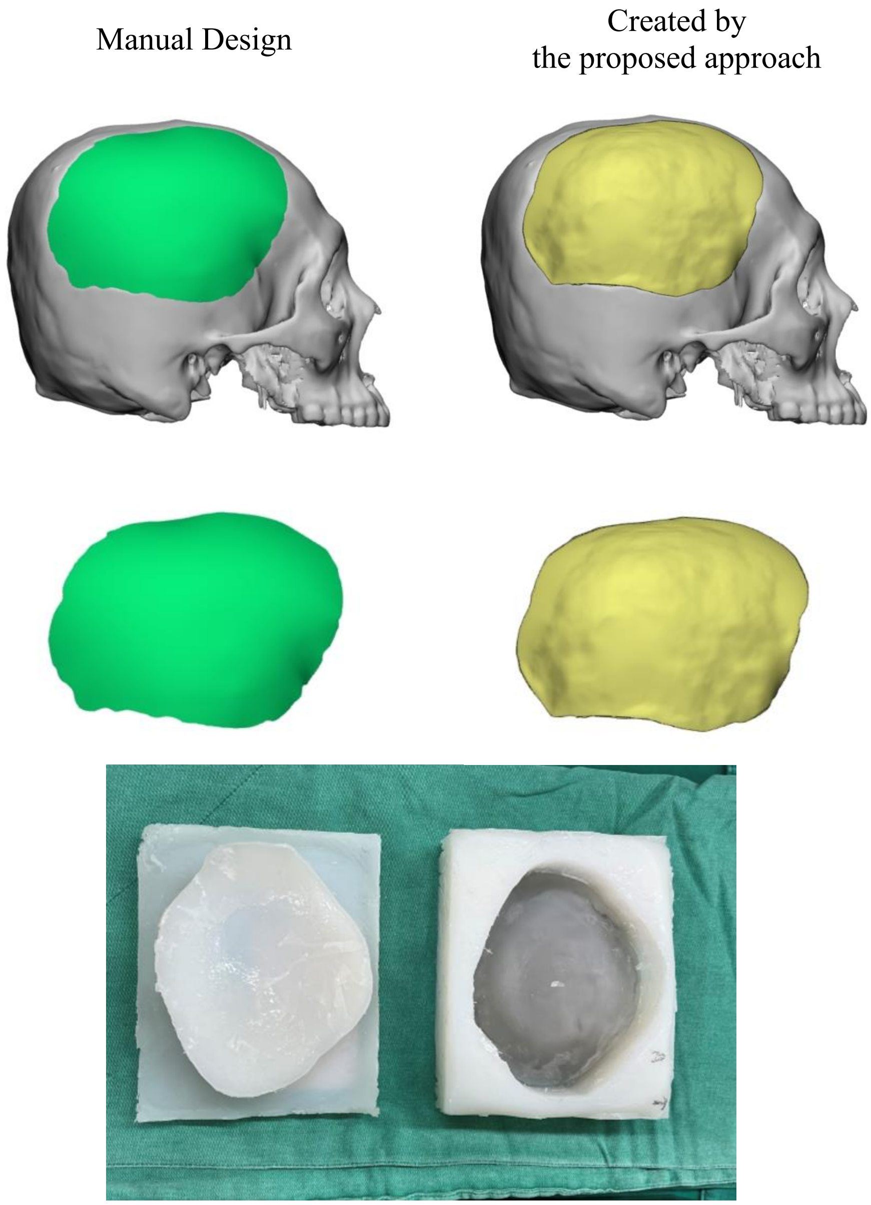

Supplementary Figure 13. More diagrams and a photo of the clinical example as presented in Fig. 4 of the article. The first row shows side views of reconstructed skulls created using technician-created implant (left, depicted in green for clarity) and the proposed approach (right, in yellow). The second row shows the corresponding implants. The last row shows the bone-cement implant (left), and the silicone rubber mold (right) used to mold the implant.

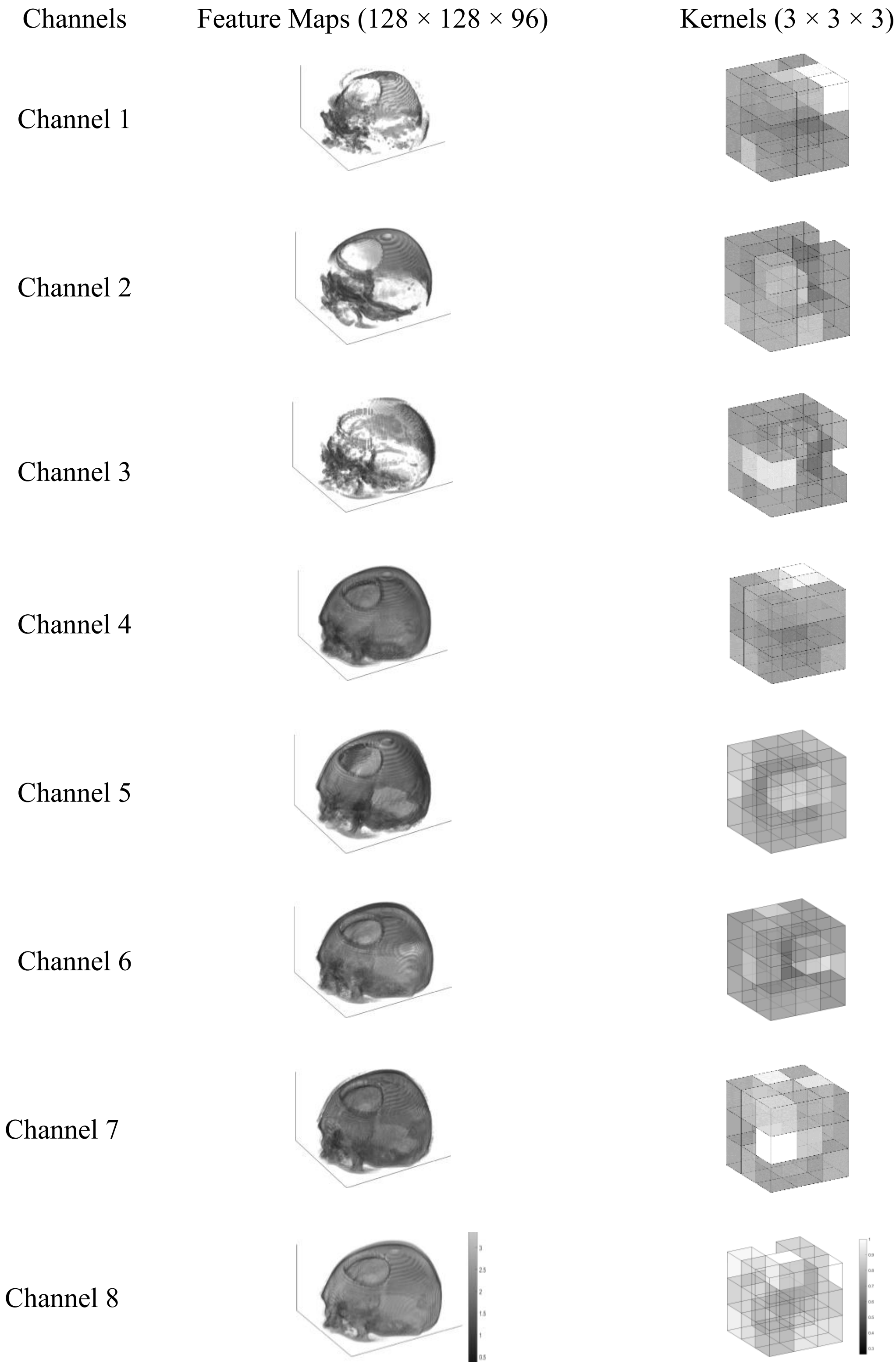

Supplementary Figure 14. Feature maps and kernel weights for the input layer of the 3D reconstruction network. The layer is denoted as conv3d_1 in Supplementary Figure 1.

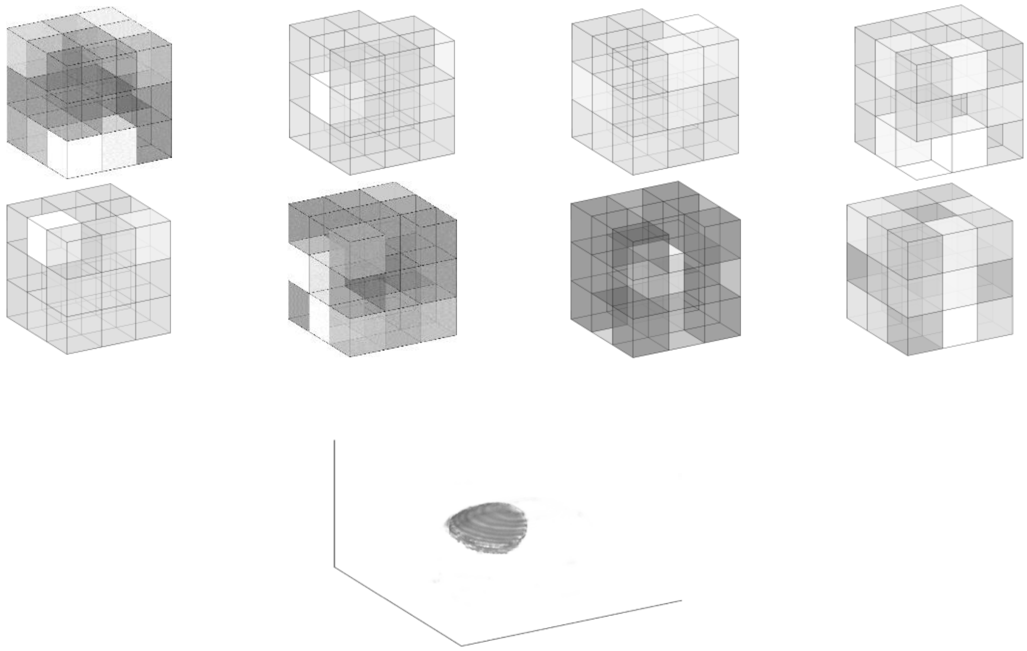

Supplementary Figure 15. Kernel weights and the feature map of the output layer in the 3D reconstruction network. The layer is denoted as conv3d_12 in Supplementary Figure 1. All kernels are of size 3 × 3 × 3, and the feature map is of size 128 × 128 × 96.

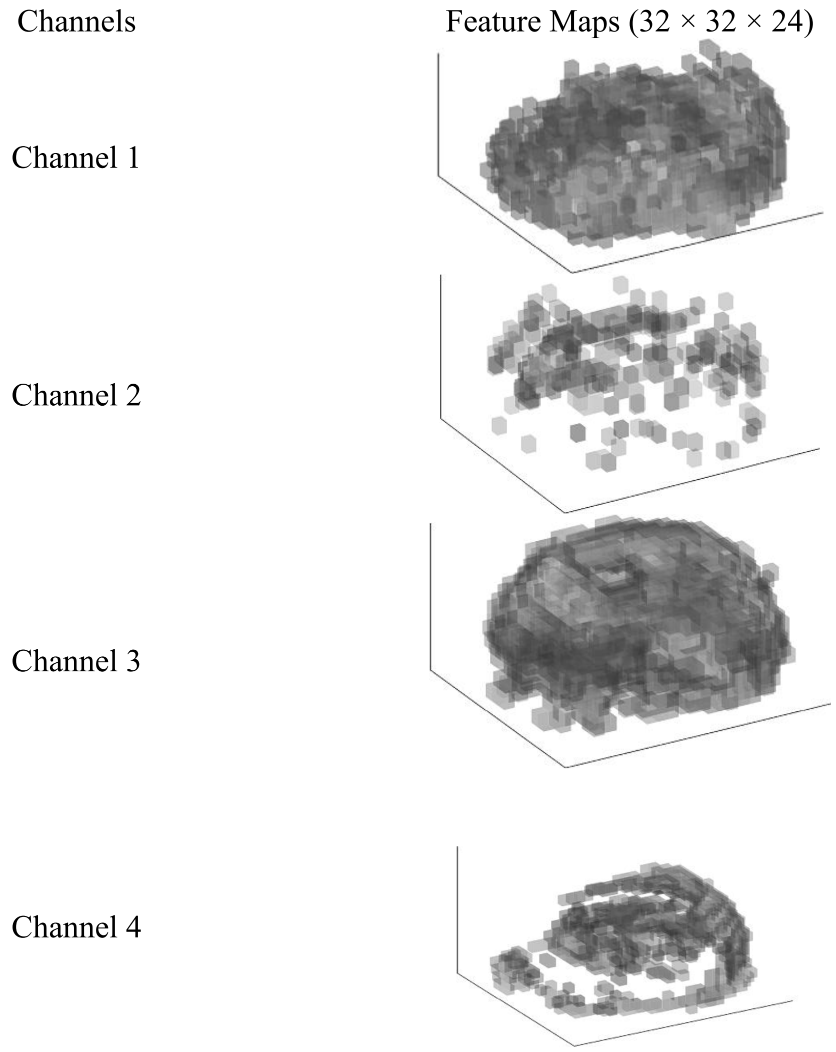

Supplementary Figure 16. Feature maps for the layer before entering the bottleneck section of the 3D reconstruction network. The layer is denoted as conv3d_4 in Supplementary Figure 1.

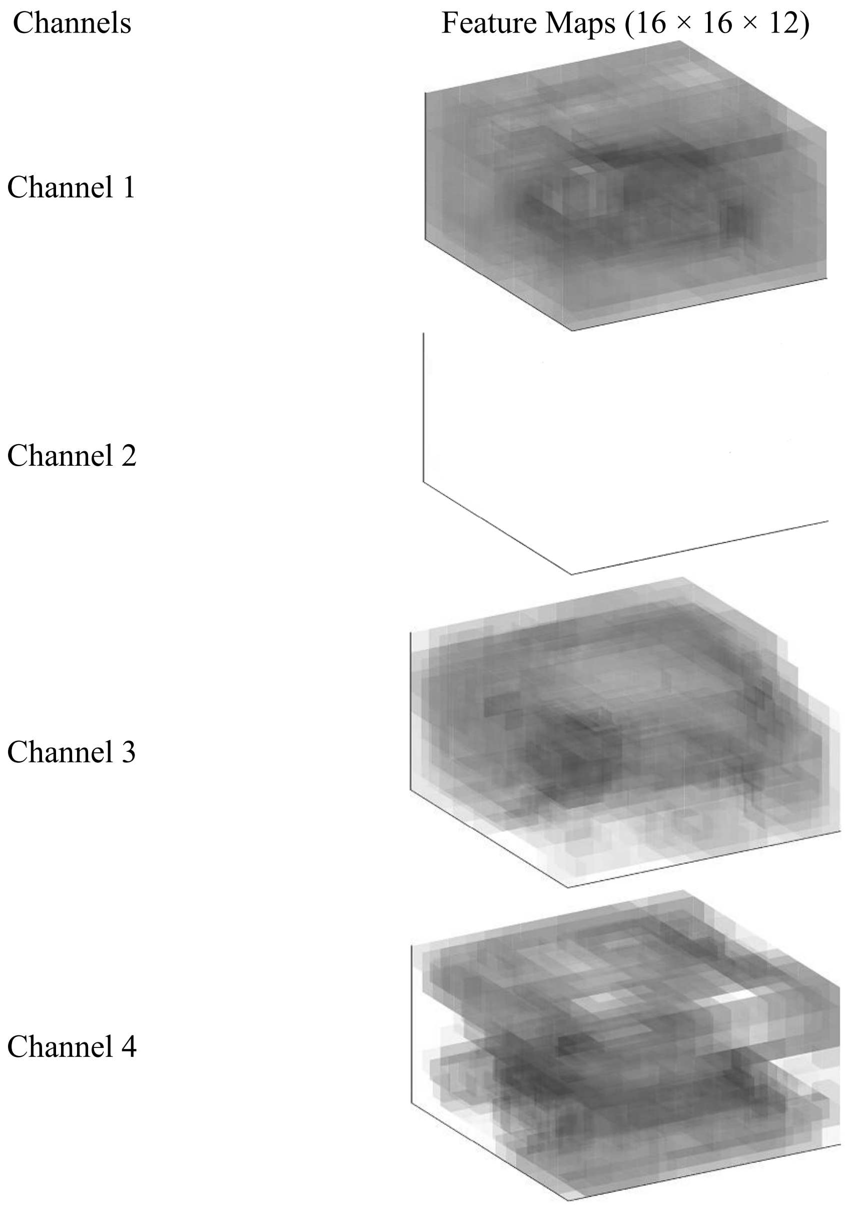

Supplementary Figure 17. Feature maps for the last layer of the bottleneck section in the 3D reconstruction network. The layer is denoted as conv3d_9 in Supplementary Figure 1.

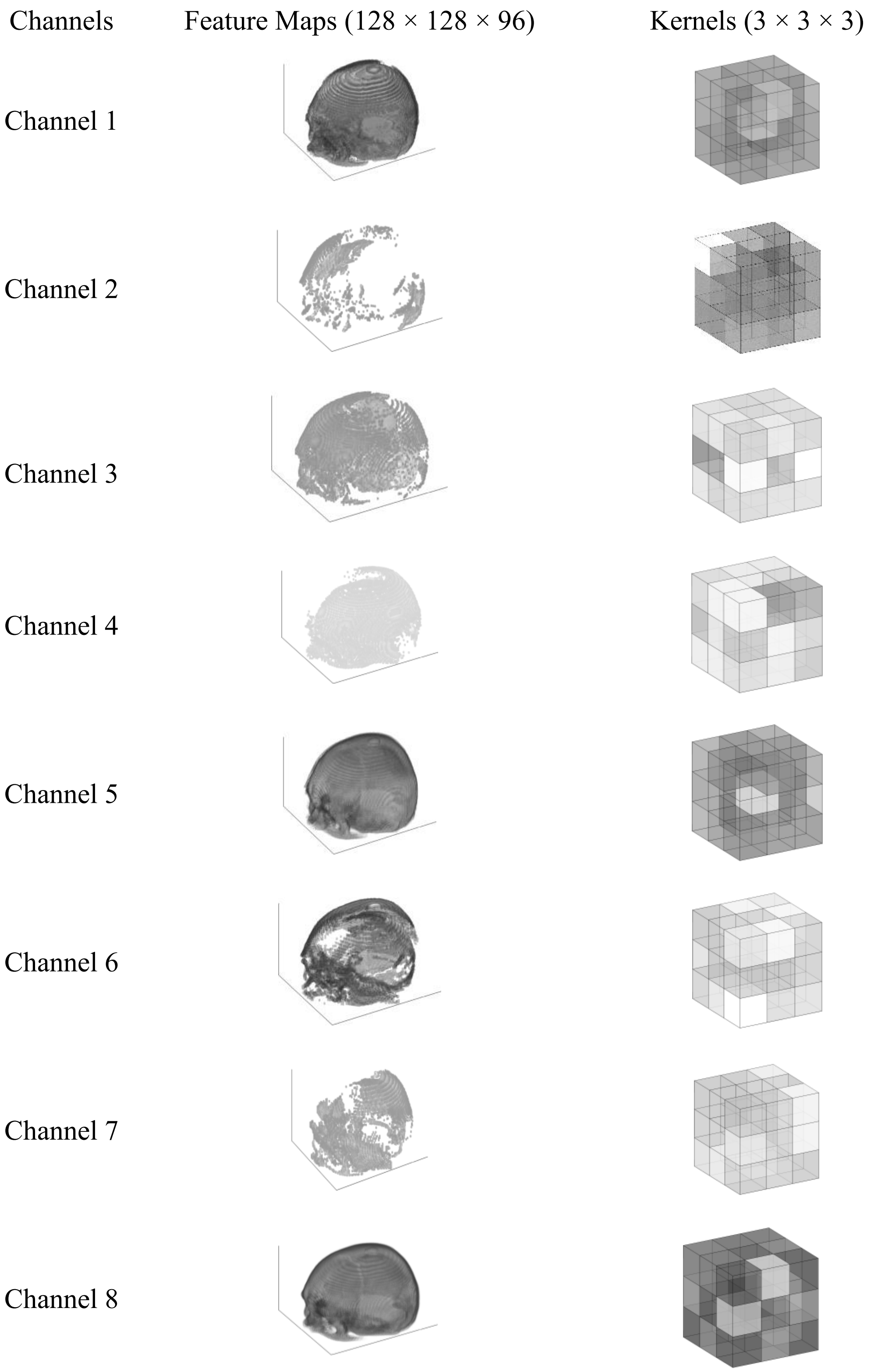

Supplementary Figure 18. Feature maps and kernel weights for the low-resolution input layer of the resolution enhancement network. The layer is denoted as conv3d_3 in Supplementary Figure 3.

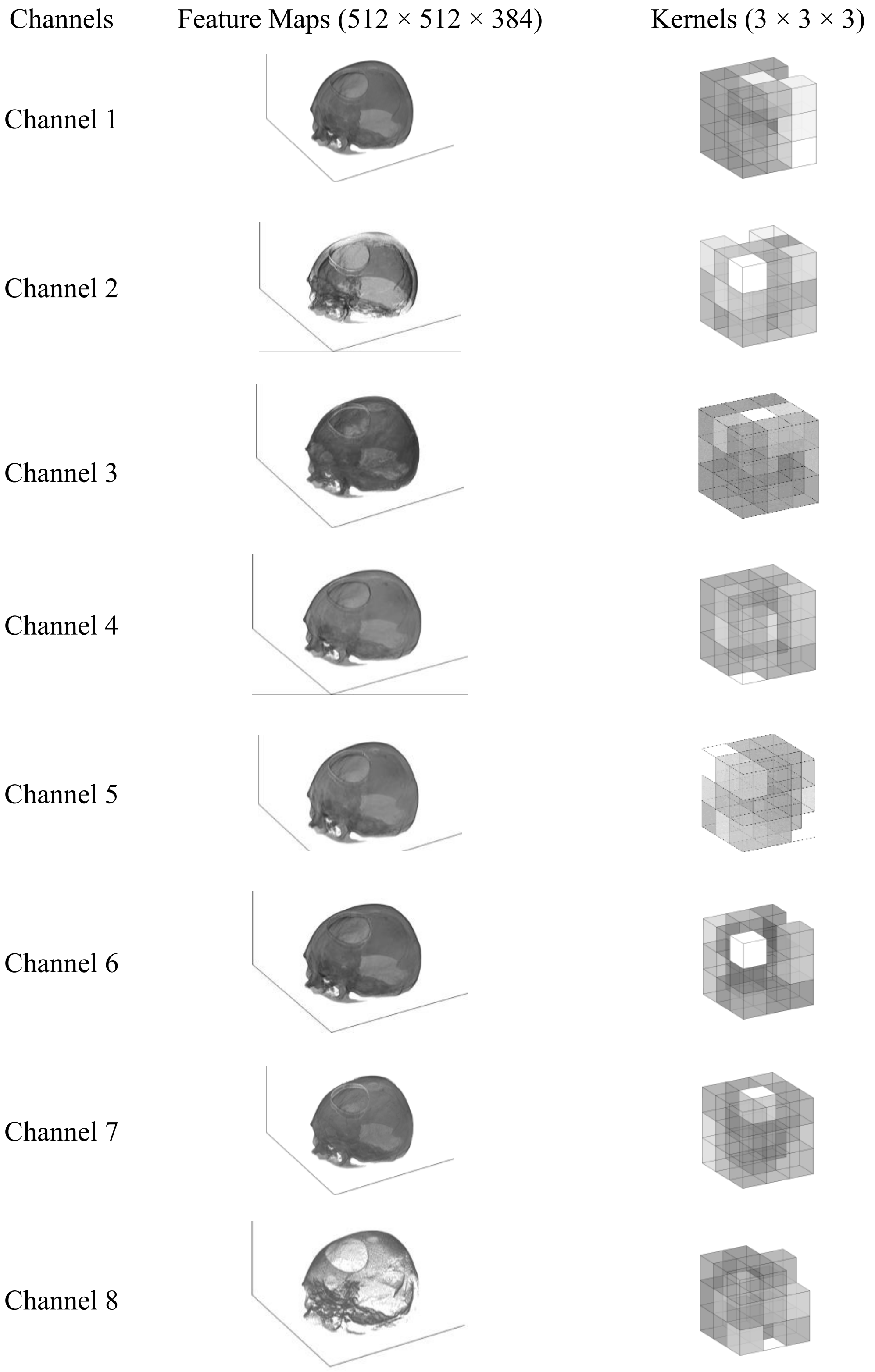

Supplementary Figure 19. Feature maps and kernel weights for the high-resolution input layer of the resolution enhancement network. The layer is denoted as conv3d_1 in Supplementary Figure 3.

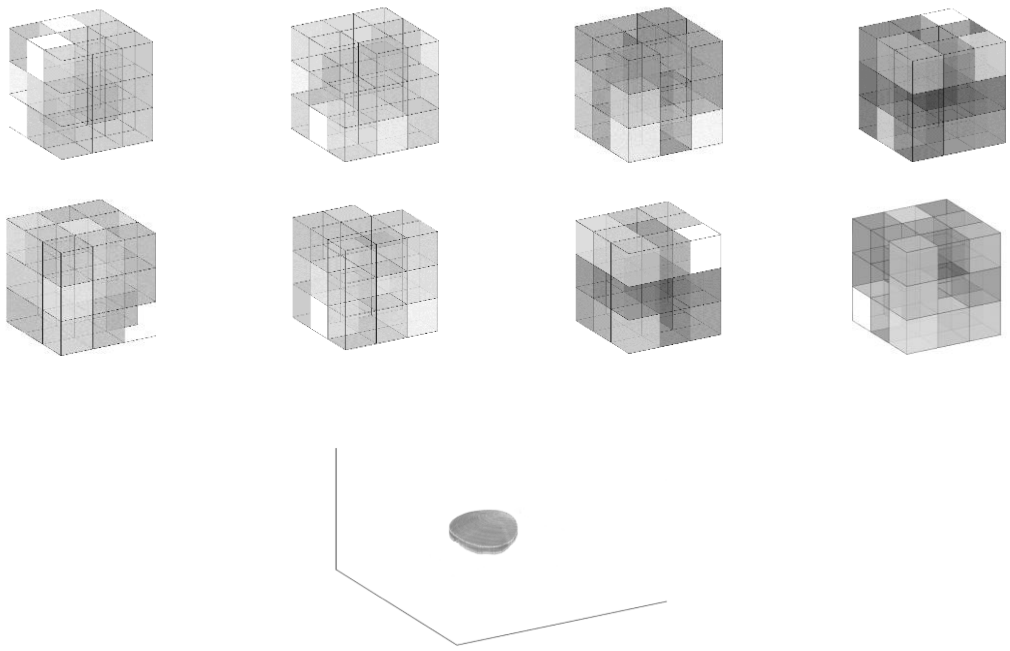

Supplementary Figure 20. Feature maps and kernel weights for the layer before the output layer of the resolution enhancement network. The layer is denoted as conv3d_16 in Supplementary Figure 3. All kernels are of size 3 × 3 × 3, and the feature map is of size 512 × 512 × 384.
